# Intravitreal corticosteroids for diabetic macular edema: a network meta-analysis of randomized controlled trials

**DOI:** 10.1186/s40662-021-00261-3

**Published:** 2021-10-11

**Authors:** Lu Gao, Xu Zhao, Lei Jiao, Luosheng Tang

**Affiliations:** 1 Department of Ophthalmology, The Second Xiangya Hospital, Central South University, 139 Middle Renmin Road, Changsha, 410011 Hunan China; 2 Department of Ophthalmology, The First Affiliated Hospital of Guangzhou Medical University, Guangzhou, China; 3 Department of Anesthesiology, The Second Xiangya Hospital, Central South University, Changsha, 410011 Hunan China; 4 School of Medicine, Taizhou University, Taizhou, Zhejiang China

**Keywords:** Diabetic macular edema, Corticosteroids, Triamcinolone acetonide, Fluocinolone acetonide, Dexamethasone, Best-corrected visual acuity, Central macular thickness, Intraocular pressure, Network meta-analysis, Randomized controlled trial

## Abstract

**Background:**

To evaluate the efficacy and safety of different intravitreal corticosteroids for treating diabetic macular edema (DME).

**Methods:**

Four databases were systematically searched for randomized controlled trials comparing different intravitreal corticosteroids for treating DME. The primary outcome was the change in best-corrected visual acuity (BCVA) within 6 months after the first injection (short-term BCVA). Secondary outcomes were the change in BCVA over 1 year (long-term BCVA) and changes in central macular thickness (CMT) and intraocular pressure (IOP) within 6 months after the first injection. Network meta-analysis was performed to aggregate the results from the individual studies.

**Results:**

Nineteen trials involving 2839 eyes were included. Intravitreal triamcinolone acetonide (TA) injections (≥ 8 mg and 4–8 mg), fluocinolone acetonide (FA) implants (0.5 µg/day) and dexamethasone (DEX) implants (700 µg) improved short-term BCVA (mean changes in logMAR [95% confidence interval] − 0.27 [− 0.40, − 0.15]; − 0.12 [− 0.18, − 0.06]; − 0.10 [− 0.21, − 0.01]; and − 0.06 [− 0.11, − 0.01]). Intravitreal TA injections (4 mg, multiple times), FA implants (0.5 µg/day and 0.2 µg/day), and DEX implants (350 µg) improved long-term BCVA (mean changes in logMAR [95% confidence interval] − 0.11 [− 0.21, − 0.02]; − 0.09 [− 0.15, − 0.03]; − 0.09 [− 0.14, − 0.02]; and − 0.04 [− 0.07, − 0.01]). All intravitreal corticosteroids reduced CMT, and different dosages of TA did not show significant differences in increasing IOP.

**Conclusions:**

Intravitreal corticosteroids effectively improved BCVA in DME patients, with higher dosages showing greater efficacies. TA was not inferior to FA or DEX and may be considered a low-cost alternative choice for DME patients. The long-term efficacy and safety of different corticosteroids deserve further investigation.

*Trial registration* Prospectively registered: PROSPERO, CRD42020219870

**Supplementary Information:**

The online version contains supplementary material available at 10.1186/s40662-021-00261-3.

## Background

Diabetic macular edema (DME) is one of the leading causes of vision loss in patients with diabetic retinopathy [1, 2]. In addition to intensive glycemic control, the management of DME requires multidisciplinary care, including intravitreal anti-vascular endothelial growth factor (VEGF) drugs, intravitreal corticosteroids, laser photocoagulation, and vitrectomy [3, 4]. Currently, intravitreal anti-VEGF drugs are mostly regarded as first-line therapy [5–7]. However, a significant number of DME patients do not respond to anti-VEGF drugs, and non-VEGF mediators are urgently needed for these patients [8].

With the increasing recognition of the role of inflammation in DME, intravitreal corticosteroids have been developed [9]. Corticosteroids can inhibit several cytokines and chemokines [9, 10], reduce retinal neovascularization and permeability [9], and have substantial anatomical and functional benefits for DME patients [11, 12]. Therefore, patients resistant to anti-VEGF drugs might respond to corticosteroids [13, 14]. Recent observational studies shed light on the effectiveness of corticosteroids for treating both naïve and refractory eyes and ameliorating the disorganization of retinal inner layers [15–17]. Three systematic reviews and meta-analyses have been performed to investigate the effect of intravitreal corticosteroids on DME patients. The first study, published in 2008, concluded that intravitreal corticosteroids might improve visual outcomes in patients with persistent or refractory DME [18]. The second study, published in 2015, concluded that slow-release corticosteroid implants are effective for treating macular edema [19]. A recent meta-analysis published in 2021 confirmed favorable visual and anatomical outcomes following fluocinolone acetonide (FA) insertion for chronic DME [20].

However, these previous pairwise meta-analyses did not compare different corticosteroids. Due to the heterogeneity among types, dosages, and pharmacokinetics, patients respond differently to different intravitreal corticosteroids [9]. While pairwise meta-analyses only estimate the effect size between two treatments based on direct evidence (head-to-head comparison of two treatments), it is challenging to determine which corticosteroid is best based on pairwise meta-analyses alone. Therefore, a network meta-analysis, which uses direct and indirect evidence (comparing two treatments via an intermediate comparator) for effect size estimation, can be applied in this scenario [21]. Furthermore, network meta-analysis can be used to rank multiple treatments, which facilitates result interpretations [22].

In this study, we performed a systematic review and network meta-analysis to assess the efficacy of different intravitreal corticosteroids for improving best-corrected visual acuity (BCVA) and reducing central macular thickness (CMT) in DME patients. Considering that side effects may be the greatest concern for the clinical application of corticosteroids [3], we also investigated intraocular pressure (IOP) to assess the safety of different corticosteroids.

## Methods

### Protocol and registration

This study was conducted and presented according to the Preferred Reporting Items for Systematic Reviews and Meta-Analyses (PRISMA) Extension Statement for Reporting of Systematic Reviews Incorporating Network Meta-analyses of Health Care Interventions (PRISMA-NMA) guidelines [23] and prospectively registered in the International Prospective Register of Systematic Reviews (PROSPERO; CRD42020219870).

### Eligibility criteria

We included randomized controlled trials that compared any intravitreal corticosteroid treatment (intravitreal injection or surgical implantation) with another intravitreal corticosteroid treatment, sham, or no treatment in patients with DME. Studies that reported changes in visual acuity, CMT, or IOP before and after treatments were included. No publication year or language restrictions were used. Both full-text articles and abstracts were eligible.

### Outcomes

The primary outcome was the change in BCVA within 6 months after the first dose of treatment (short-term visual acuity). Visual acuity can be measured in the Early Treatment Diabetic Retinopathy Study (ETDRS) letter, Snellen line, or logarithm of the minimal angle of resolution (logMAR). The secondary outcomes were the change in BCVA at least 1 year after the first dose of treatment (long-term visual acuity) and changes in CMT and IOP within 6 months after the first dose of treatment (short-term CMT and IOP).

### Information sources and search

Ovid MEDLINE, Embase, Web of Science, and the Cochrane Central Register of Controlled Trials (containing the Cochrane Eyes and Vision Group Trials Register) were systematically searched for articles published from the dates of inception to November 2020. The search strategy was constructed by analyzing Medical Subject Heading (MeSH) words from relevant studies [24] and referring to a previous systematic review [18]. In summary, the strategy included three concepts: DME, corticosteroids, and randomized controlled trials. The complete search strategy and the search results in each step are presented in Additional file 1. The reference lists of all relevant articles were also screened to identify additional articles.

### Study selection and data collection

EndNote (Version 9.0) was used to screen and select eligible studies. Two investigators (LG and XZ) independently screened all nonduplicate titles and abstracts identified in the systematic search and then evaluated the full texts of the candidate articles to determine their eligibility based on the criteria described above. Disagreements were resolved by consulting a senior author (LT). The same investigators (LG and XZ) performed data extraction independently using a predesigned data form. Investigators were blinded to the results of the analyses during the study selection and data extraction process. The interrater reliability between two investigators was quantified by Cohen’s kappa [25].

### Data items and data processing

The data items extracted from each eligible study were as follows: (1) author and year, (2) patient population characteristics (region, number of centers, patient number, and age), (3) intervention characteristics (type and dosage of corticosteroids), and (4) outcome and results. If a study reported visual acuity in the ETDRS letter or Snellen line format, the results were converted into the logMAR format [26, 27]. If a study reported results at different time points (e.g., 2 and 3 years after treatment), we used the result corresponding to the longest follow-up period. If a study did not report the standard deviations for outcome measures, we imputed the standard deviations using the methods described by the Cochrane Handbook for Systematic Reviews of Interventions (Version 5.1.0, Part 3, Chapter 16.1.3.2) [28].

### Risk of bias in individual studies

We used the Cochrane Collaboration tool to assess the risk of bias in each study [29].

### Network geometry

We created geometric networks to visualize the comparisons between different types and dosages of intravitreal corticosteroids. The geometric network had different nodes corresponding to different treatments. The size of the node was determined by the total number of patients receiving a specific treatment. The nodes were connected by lines representing the number of direct comparisons.

### Summary measures

We used the mean difference, 95% confidence interval (CI), and 95% credible interval (CrI) to compare the effects of different treatments. The overall treatment ranks are presented as the surface under the cumulative ranking curve (SUCRA) scores, which range from 0 to 100% and are used to evaluate which treatment in a network is likely to be the most efficacious [30].

### Planned methods of analysis

For the network meta-analysis, we used Bayesian hierarchical random-effects models with the Markov chain Monte Carlo method to derive the pooled estimates [31]. Three chains of 100,000 iterations were used after a burn-in period of 50,000 iterations, and the initial iterations were discarded to ensure that the final estimates were based on stable posterior sampling. Trace plots and the Brooks–Gelman–Rubin statistic were used to assess convergence. The I^2^ statistic for network meta-analysis was used to evaluate the statistical heterogeneity in the network [32]. Network meta-analysis was performed using the R package “gemtc” (R version 3.5.3, R Foundation for Statistical Computing, Vienna, Austria) [33].

For direct comparisons, we synthesized the results using pairwise meta-analysis with fixed-effects models if the number of studies was less than four [34]. Otherwise, random-effects models were used. The I^2^ statistic was calculated to measure the level of heterogeneity of the included studies. The pairwise meta-analysis was performed using the R package “metaphor” (R version 3.5.3, R Foundation for Statistical Computing, Vienna, Austria) [35].

### Assessment of inconsistency, publication bias, and quality of evidence

The node splitting method was used to calculate the inconsistency between the direct and indirect comparisons [36]. Funnel plots and Egger’s test were used to analyze the potential publication bias for direct comparisons of three or more studies [37]. The quality of evidence was assessed according to the Grading of Recommendations Assessment, Development, and Evaluation (GRADE) guidelines [38].

## Results

### Study selection

**Fig. 1:**
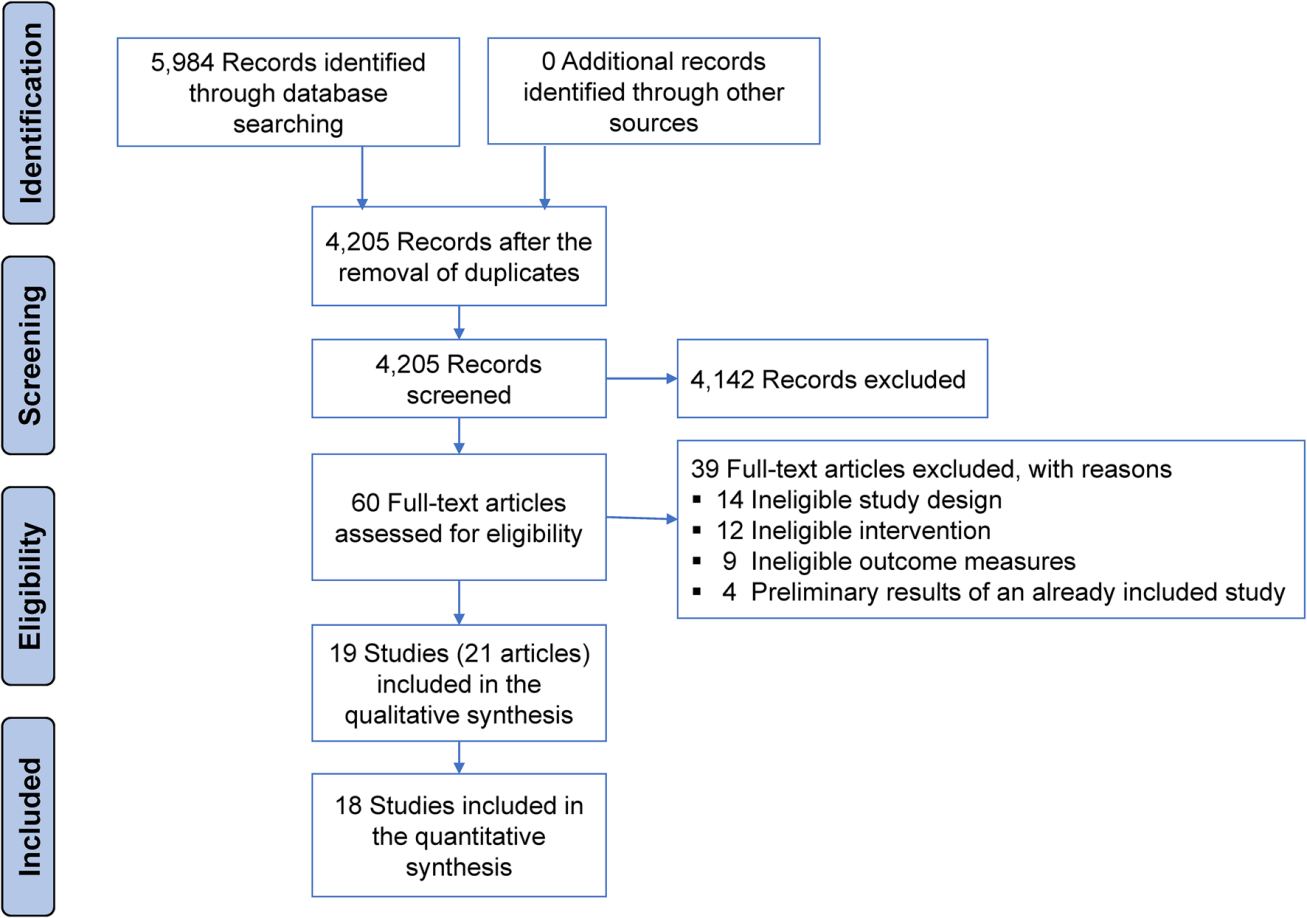
Flow diagram of the systematic literature search and study selection processes

The study selection process is presented in Fig. 1. Of the 4205 nonduplicate records screened, we identified 21 eligible studies [39–59]. Because two of these studies reported the 3-year outcomes of previous randomized controlled trials [54, 57], a total of 19 randomized controlled trials were included. There was 95% agreement between investigators (LG and XZ) for study inclusion (Cohen’s kappa = 0.81).

### Characteristics of the individual studies

The characteristics of the included studies are presented in Table 1, and the details of the outcome measures are presented in Additional file 1: Table S1. A total of 2839 DME eyes were investigated. The included patients were treatment-naïve or had persistent DME for at least 3 months after laser photocoagulation treatment, anti-VEGF treatment or another medical treatment. The mean age of these patients ranged from 53 to 72 years. Three types of corticosteroids, including triamcinolone acetonide (TA), FA, and dexamethasone (DEX), were investigated. TA was given by an intravitreal injection at doses of 1 mg, 2 mg, 4 mg, 5 mg, 8 mg, 13 mg, or 20 mg per injection. FA was given by surgical implantation at dosages of 0.2 µg/day or 0.5 µg/day. DEX was given by intravitreal injection or surgical implantation, with total dosages ranging from 350 to 800 µg. Since several TA dosages were used, we divided the patients who received TA treatments into three groups (i.e., intravitreal TA injections < 4 mg, 4–8 mg, and ≥ 8 mg) to facilitate analysis and interpretation (Table 1). Among the eligible trials, 16 reported short-term changes in BCVA, 4 reported long-term changes in BCVA, 12 reported short-term changes in CMT, and 6 reported short-term changes in IOP (Table 1). For the CMT measurement, seven trials were based on time-domain optical coherence tomography (OCT), one on spectral-domain OCT, one on a retinal thickness analyzer, and three without device information (Additional file 1: Table S1).

**Table 1:** Characteristics of the included studies

First author (year)	Design (region)	Age^a^ (years)	Intervention description	Intervention category	Number of eyes	Outcome used in network meta-analysis^b^
Sutter (2004) [39]	Single-center (Australia)	64	Intravitreal injection of 4 mg TA once	TA injection (4–8 mg)	33	3-month BCVA change (short-term) 3-month CMT change (short-term)
			Subconjunctival injection of saline once	Control	32	
Spandau (2005) [40]	Single-center (Germany)	70	Intravitreal injection of 2 mg TA once	TA injection (< 4 mg)	8	6-month BCVA change (short-term) IOP change at study end (mean follow-up = 6.6 months, short-term)^c^
			Intravitreal injection of 5 mg TA once	TA injection (4–8 mg)	10	
			Intravitreal injection of 13 mg TA once	TA injection (≥ 8 mg)	9	
Audren (2006) [41]	Single-center (France)	60	Intravitreal injection of 4 mg TA once	TA injection (4–8 mg)	17	6-month BCVA change (short-term) 6-month CMT change (short-term) 6-month IOP change (short-term)
			No injection	Control	17	
Audren (2006) [42]	Single-center (France)	64	Intravitreal injection of 2 mg TA once	TA injection (< 4 mg)	16	6-month BCVA change (short-term) 6-month CMT change (short-term) 6-month IOP change (short-term)
			Intravitreal injection of 4 mg TA once	TA injection (4–8 mg)	16	
Gillies (2006) [43]	Single-center (Australia)	64	Intravitreal injection of 4 mg TA multiple times (median times of treatment = 3)	TA injection (4 mg, multiple times)	31	2-year BCVA change (long-term)
			Subconjunctival injection of saline multiple times	Control	29	
Jonas (2006) [44]	Single-center (Germany)	66	Intravitreal injection of 20 mg TA once	TA injection (≥ 8 mg)	28	BCVA change at study end (mean follow-up = 10 months, short-term)^c^ IOP change at study end (mean follow-up = 10 months, short-term)^c^
			No injection	Control	12	
Lam (2007) [45]^d^	Multicenter (China)	65	Intravitreal injection of 4 mg TA once	TA injection (4–8 mg)	23	26-week BCVA change (short-term) 26-week CMT change (short-term)
			Intravitreal injection of 8 mg TA once	TA injection (≥ 8 mg)	20	
Dehghan (2008) [46]	Single-center (Iran)	62	Intravitreal injection of 4 mg TA once	TA injection (4–8 mg)	42	4-month BCVA change (short-term) 4-month CMT change (short-term)
			Subconjunctival injection of 2% lidocaine once	Control	37	
Hauser (2008) [47]^e^	Single-center (Israel)	67	Intravitreal injection of 2 mg TA once	TA injection (< 4 mg)	17	6-month BCVA change (short-term) 6-month CMT change (short-term) 6-month IOP change (short-term)
			Intravitreal injection of 4 mg TA once	TA injection (4–8 mg)	13	
Kim (2008) [48]	Multicenter (United States)	61	Intravitreal injection of 2 mg TA once	TA injection (< 4 mg)	13	6-month BCVA change (short-term)
			Intravitreal injection of 4 mg TA once	TA injection (4–8 mg)	15	
Larsson (2009) [49]	Single-center (Australia)	62	Intravitreal injection of 4 mg TA once	TA injection (4–8 mg)	16	3-month BCVA change (short-term)
			Subconjunctival injection of saline once	Control	16	
Campochiaro (2010) [50]	Multicenter (United States)	67	Intravitreal insertion of FA implant (0.2 µg/day)	FA implant (0.2 µg/day)	20	6-month BCVA change (short-term) 1-year BCVA change (long-term) 6-month CMT change (short-term) 6-month IOP change (short-term)
			Intravitreal insertion of FA implant (0.5 µg/day)	FA implant (0.5 µg/day)	17	
Chan (2010) [51]	Multicenter (China)	67	Intravitreal injection of 400 µg DEX once	DEX injection (400 µg)	6	Not included in network meta-analysis due to the unique intervention that cannot connect with other treatments
			Intravitreal injection of 800 µg DEX once	DEX injection (800 µg)	6	
Campochiaro (2011, 2012) [52, 54]	Multicenter (Worldwide)	63	Intravitreal insertion of FA implant (0.2 µg/day)	FA implant (0.2 µg/day)	375	6-month BCVA change (short-term) 3-year BCVA change (long-term) 6-month CMT change (short-term)
			Intravitreal insertion of FA implant (0.5 µg/day)	FA implant (0.5 µg/day)	393	
			Sham injection	Control	185	
Pearson (2011) [53]	Multicenter (United States)	65	Intravitreal insertion of FA implant (0.59 µg/day)	FA implant (0.5 µg/day)	127	6-month CMT change (short-term)
			Standard of care	Control	69	
Boyer (2014) and Danis (2016) [55, 57]	Multicenter (Worldwide)	62	Intravitreal insertion of DEX implant (350 µg)	DEX implant (350 µg)	347	6-month BCVA change (short-term) 3-year BCVA change (long-term) 6-month CMT change (short-term)
			Intravitreal insertion of DEX implant (700 µg)	DEX implant (700 µg)	351	
			Sham injection	Control	350	
Lodhi (2015) [56]	Single-center (India)	55	Intravitreal injection of 1 mg TA once	TA injection (< 4 mg)	20	6-month BCVA change (short-term) 6-month IOP change (short-term)
			Intravitreal injection of 4 mg TA once	TA injection (4–8 mg)	20	
Mylonas (2016) [58]	Multicenter (Europe)	72	Intravitreal injection of 4 mg TA once	TA injection (4–8 mg)	14	6-month BCVA change (short-term) 6-month CMT change (short-term)
			Intravitreal insertion of DEX implant (700 µg)	DEX implant (700 µg)	15	
Zhou (2016) [59]	Single-center (China)	53	Intravitreal injection of 2 mg TA once	TA injection (< 4 mg)	27	6-month BCVA change (short-term) 6-month CMT change (short-term)
			Intravitreal injection of 4 mg TA once	TA injection (4–8 mg)	27	

*TA* triamcinolone acetonide; *BCVA* best-corrected visual acuity; *CMT* central macular thickness; *IOP* intraocular pressure; *FA* fluocinolone acetonide; *DEX* dexamethasone

^a^Mean or median age of the enrolled patients in the studies

^b^The time listed in this column (3-month, 6-month, etc.) corresponds to the time of the outcome assessment after the first intravitreal corticosteroid injection/implantation

^c^These outcomes were assessed at the study end. We regarded them as short-term outcomes, considering that the mean follow-up time was less than 1 year

^d^In this study, we did not include the group with 6 mg TA because the baseline BCVA of this group was significantly worse than that of the 4 mg and 8 mg groups

^e^In this study, we did not include the group with 1 mg TA because the baseline BCVA of this group was significantly worse than that of the 2 mg and 4 mg groups

### Risk of bias within studies

The risks of bias within individual studies are presented in Additional file 1: Table S2. A total of 71% of the studies had a low risk of bias, 19% had an unclear risk of bias, and 10% had a high risk of bias. The pooled risk of bias is presented in Additional file 1: Figure S1.

### Short-term BCVA

**Fig. 2:**
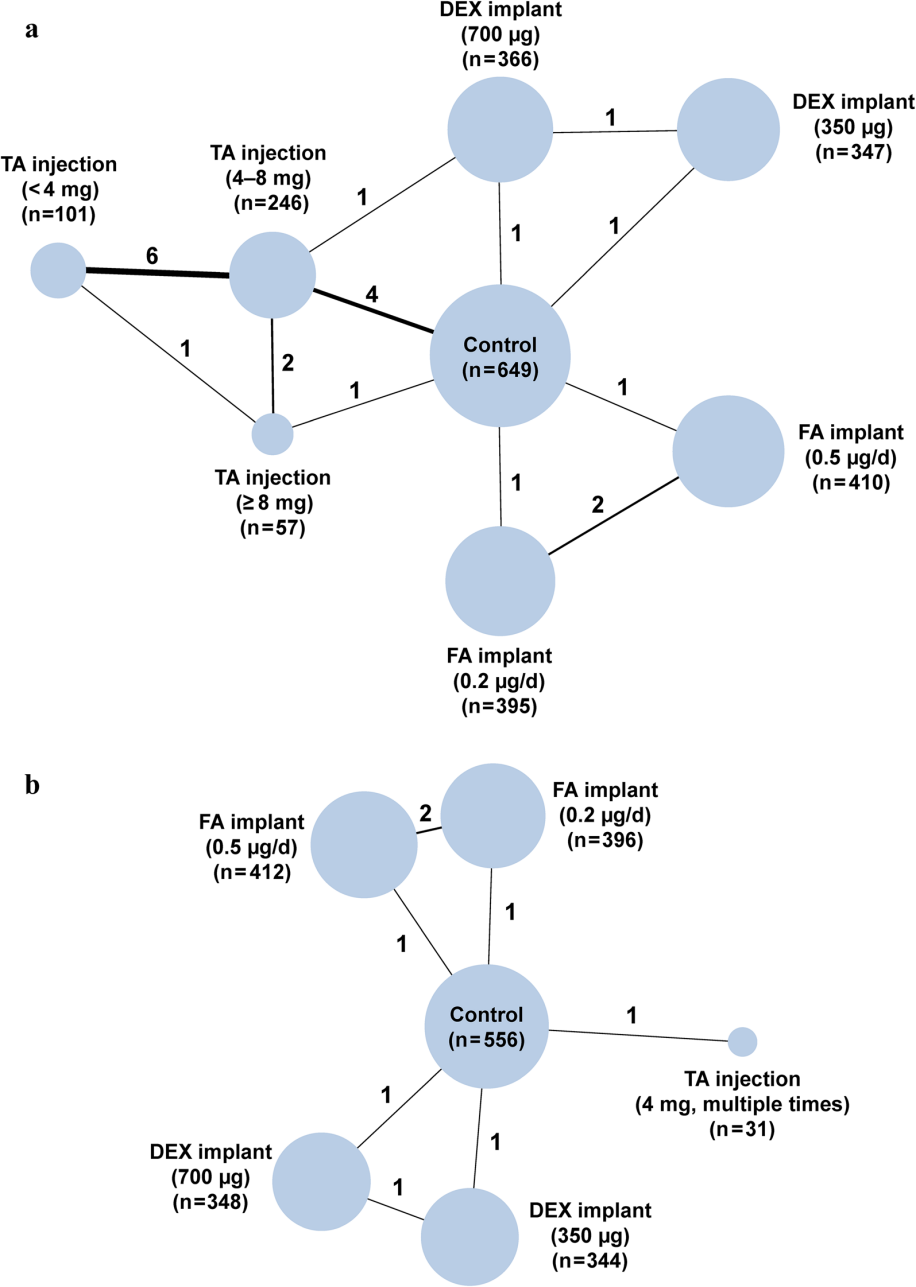
Network geometry of different intravitreal corticosteroids to improve **a** short-term and **b** long-term BCVA. *BCVA,* best-corrected visual acuity; *TA,* triamcinolone acetonide; *FA,* fluocinolone acetonide; *DEX*, dexamethasone

**Fig. 3:**
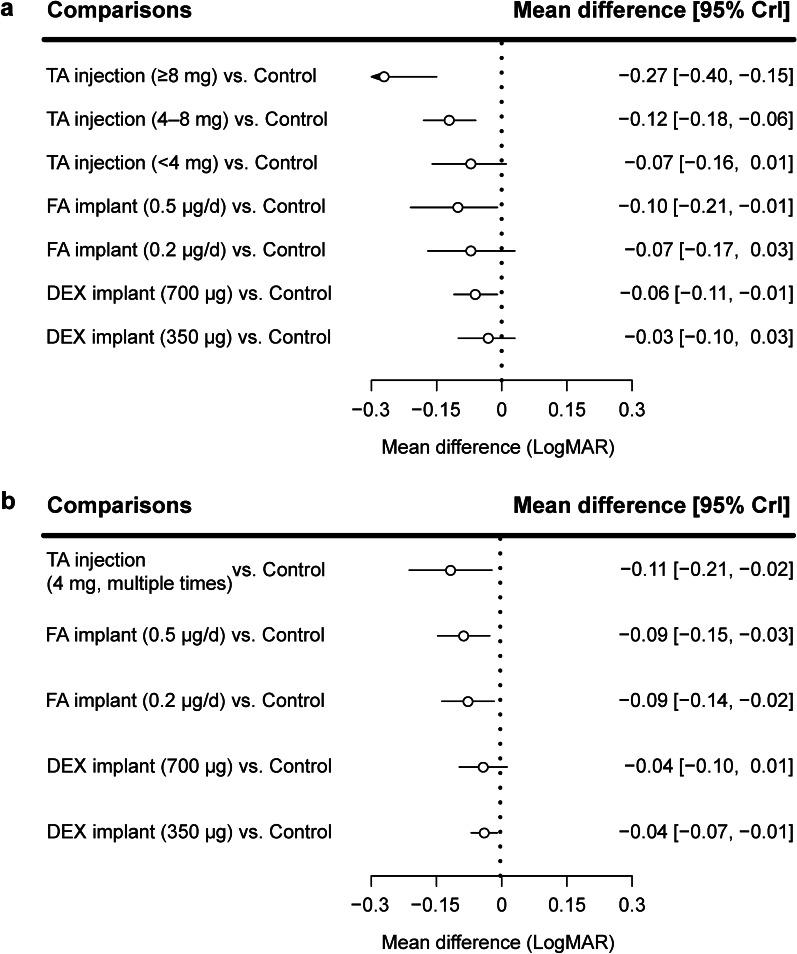
Effects of the different intravitreal corticosteroids on **a** short-term and **b** long-term BCVA. *BCVA,* best-corrected visual acuity; *CrI,* credible interval; *TA*, triamcinolone acetonide; *FA,* fluocinolone acetonide; *DEX*, dexamethasone

**Fig. 4:**
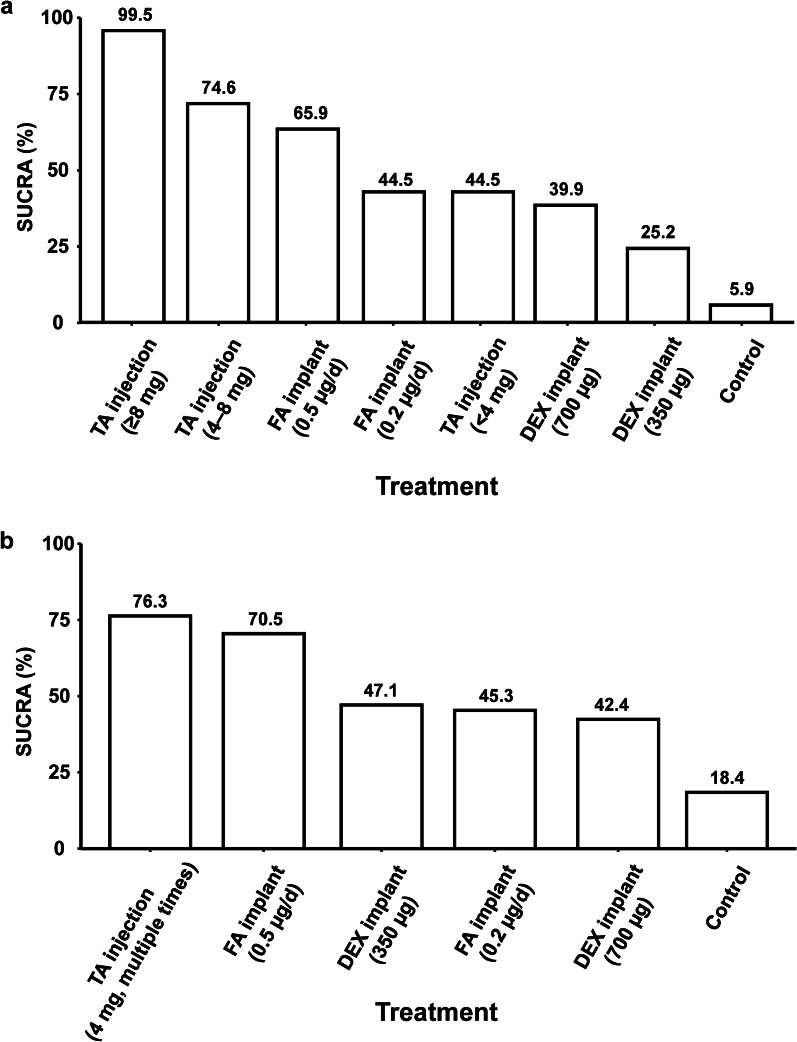
SUCRA scores for the effects of different intravitreal corticosteroids on **a** short-term and **b** long-term BCVA. *SUCRA*, surface under the cumulative ranking curve; *BCVA*, best-corrected visual acuity; *TA*, triamcinolone acetonide; *FA*, fluocinolone acetonide; *DEX*, dexamethasone

A total of 35 randomization groups from 16 trials were assigned to 8 nodes corresponding to different treatments (Fig. 2a). The results and evidence quality assessments are presented in Fig. 3a and Additional file 1: Tables S3–S5. The statistical heterogeneity (I^2^) in this network was 4%. Compared to sham or no treatment (control), intravitreal TA injections ≥ 8 mg (mean difference in logMAR [95% CrI], − 0.27 [− 0.40, − 0.15]; low quality), TA injections of 4–8 mg (mean difference in logMAR [95% CrI], − 0.12 [− 0.18, − 0.06]; high quality), FA implants of 0.5 µg/day (mean difference in logMAR [95% CrI], − 0.10 [− 0.21, − 0.01]; moderate quality), and DEX implants of 700 µg (mean difference in logMAR [95% CrI], − 0.06 [− 0.11, − 0.01]; moderate quality) improved short-term BCVA (Fig. 3a). Intravitreal TA injections ≥ 8 mg showed larger improvements in short-term BCVA than did other treatments (mean difference from − 0.24 to − 0.16; very low or low quality; Additional file 1: Table S3). Intravitreal TA injections ≥ 8 mg, TA injections of 4–8 mg, and FA implants of 0.5 µg/day were likely the most efficacious for improving short-term BCVA (SUCRA = 99.5%, 74.6%, and 65.9%, respectively; Fig. 4a). There was no publication bias (Additional file 1: Table S4) or inconsistency between the direct and indirect comparisons (Additional file 1: Table S5).

### Long-term BCVA

A total of 10 randomization groups from 4 trials were assigned to 6 nodes corresponding to different treatments (Fig. 2b). The results and evidence quality assessments are presented in Fig. 3b and Additional file 1: Tables S6, S7. The statistical heterogeneity (I^2^) in this network was 19%. Compared to the control condition, multiple intravitreal TA injections of 4 mg (mean difference in logMAR [95% CrI], − 0.11 [− 0.21, − 0.02]; low quality), FA implants of 0.5 µg/day (mean difference in logMAR [95% CrI], − 0.09 [− 0.15, − 0.03]; moderate quality), FA implants of 0.2 µg/day (mean difference in logMAR [95% CrI], − 0.09 [− 0.14, − 0.02]; moderate quality), and DEX implants of 350 µg (mean difference in logMAR [95% CrI], − 0.04 [− 0.07, − 0.01]; moderate quality) improved long-term BCVA (Fig. 3b). There were no differences among these treatments (low or moderate quality; Additional file 1: Table S6). Multiple intravitreal TA injections of 4 mg and FA implants of 0.5 µg/day were likely the most efficacious for improving long-term BCVA (SUCRA = 76.3% and 70.5%, respectively; Fig. 4b). There was no publication bias (Additional file 1: Table S4). We did not explore inconsistency because no comparisons included both direct and indirect evidence.

### Short-term CMT and IOP

A total of 26 randomization groups from 12 trials were assigned to 8 nodes corresponding to different treatments (Additional file 1: Figure S2). The statistical heterogeneity (I^2^) in this network was 9%. Compared to the control condition, all intravitreal corticosteroid treatments reduced CMT in the short term (Additional file 1: Figure S3). The details related to the effects of different intravitreal corticosteroids in reducing CMT are presented in Additional file 1: Tables S8–S10. Intravitreal TA injections ≥ 8 mg and FA implants of 0.5 µg/day were likely the most efficacious for reducing short-term CMT (SUCRA = 93.0% and 80.6%, respectively; Additional file 1: Figure S4).

Changes in IOP were reported by six studies, in which only intravitreal TA injections and the control condition were investigated (Additional file 1: Figure S5). The statistical heterogeneity (I^2^) in this network was 16%. Compared to the control condition, intravitreal TA injections ≥ 8 mg (mean difference in mmHg [95% CrI], 2.08 [− 1.05, 5.52]; low quality), TA injections of 4–8 mg (mean difference in mmHg [95% CrI], 2.38 [− 0.75, 5.70]; low quality), and TA injections < 4 mg (mean difference in mmHg [95% CrI], 1.92 [− 1.58, 5.64]; low quality) did not show significant differences in increasing IOP (Additional file 1: Figure S6 and Tables S11–S13).

## Discussion

### Summary of the evidence

This is the first network meta-analysis comparing the efficacy and safety of different intravitreal corticosteroids for treating patients with DME. In this study, we included 19 eligible randomized controlled trials involving 2839 DME eyes. The results showed that intravitreal TA injections, FA implants, and DEX implants could improve BCVA in both the short and long term in patients with DME. Higher dosages of corticosteroids (TA ≥ 4 mg, FA implant of 0.5 µg/day, and DEX implant of 700 µg) showed greater levels of efficacy for improving BCVA within 6 months after treatment. Intravitreal TA injections (≥ 8 mg) were possibly the most efficacious for improving BCVA within 6 months after the first injection. All intravitreal corticosteroids reduced the CMT. However, data about IOP change were only available for intravitreal TA injections, and different dosages of TA did not show a significant difference in IOP increases.

According to the European Society of Retina Specialists (EURETINA) guidelines, corticosteroids are recommended as a second choice for treating DME patients, especially for non-responders who have been treated with anti-VEGF drugs [60]. Among the three types of intravitreal corticosteroid management (i.e., TA injections, FA implants, and DEX implants), DEX is recommended to be used first, while FA might be used for patients who are not responsive to other corticosteroids [60]. Due to the side effects of TA, this drug should only be considered when DEX and FA are unavailable [60]. The above recommendations are mainly based on the results of several high-quality trials, which compared corticosteroids with control or other types of treatments [52, 55, 61]. However, different corticosteroids were not directly compared in a single study to explore their relative efficacy. Meanwhile, in view of the high cost of DEX and FA implants, especially for lower-middle-income countries, evaluating the value of intravitreal TA as a low-cost alternative for DME patients is still worthwhile [62].

The novel findings of our study can be summarized as follows: (1) all types of corticosteroids improved visual outcomes for DME patients, with higher dosages (TA ≥ 4 mg, FA implant of 0.5 µg/day, and DEX implant of 700 µg) showing greater levels of improvements in BCVA; (2) intravitreal TA was not inferior to FA or DEX for improving BCVA or decreasing CMT in DME patients. A large dosage of intravitreal TA injections (≥ 8 mg) provided a greater improvement in BCVA than other dosages of TA, FA, and DEX. These results suggest that TA can be considered for DME patients requiring corticosteroid treatments; (3) DEX and FA implants were comparable for improving visual outcomes in DME patients; and (4) different dosages of TA did not show significant differences to increases in IOP. However, due to the low quality of evidence, we could not rule out the risk of IOP increase after TA injections; (5) although we cannot definitively conclude that one corticosteroid is better than the rest, we have comprehensively summarized the types and dosages of intravitreal corticosteroids in randomized controlled trials. Our study also highlights the persistent gap in the literature of high-quality evidence regarding intravitreal corticosteroids.

The differences in corticosteroid efficacy, safety, and administration routes can be partly explained by their pharmacokinetics. More water-soluble corticosteroids can improve drug loading but decrease the half-life of the drug in the vitreous [9]. The water solubility of TA is only 20% that of DEX, leading to an extended presence in the vitreous (mean elimination half-life of 18.6 days) [9, 63]. Thus, TA can be used without a sustained-release delivery system. Instead, DEX and FA are highly water soluble and require sustained-release delivery systems to maintain prolonged drug levels in the vitreous [9]. Although a single injection of TA can meet the short-term clinical requirement for treating DME, the maximum dose and the maximum duration of drug release are limited [9]. This is why in Gillies et al.’s study (the only study investigating the effects of TA on 2-year outcomes), patients received repeated TA injections (1–5 injections) to maintain TA potency [43]. Regarding the FA and DEX implants, although the potency of DEX is fivefold higher than that of TA, FA has a longer durability than DEX [9]. The DEX implant releases the corticosteroid into the vitreous over a period of ≤ 6 months [64], while the FA implant provides sustained delivery in the eye for at least 1 year [50]. Our study did not find a significant difference between FA and DEX’s efficacies for visual outcomes. However, FA may be more suitable for long-term treatments because it reduces the number of repeated interventions.

In addition to efficacy, side effects are crucial when assessing the best type of intravitreal corticosteroids for DME patients. The increased IOP and risk of glaucoma are the biggest concerns for intravitreal corticosteroids [3]. Corticosteroid-induced IOP elevation depends on corticosteroid potency, pharmacokinetics, duration of treatment, and administration route [65]. While the volume of the vitreous cavity is relatively fixed (approximately 4 ml), intravitreal corticosteroid injections (approximately 0.1 ml) can immediately increase IOP after injection because they cause an approximately 2.5% increase in the volume of fluids in the vitreous cavity [66]. However, the situation with intravitreal corticosteroid implants was different. There was no cumulative effect of DEX implants on IOP, as the incidence of IOP elevation and the amplitude of the IOP rise did not increase after multiple implants [55]. In this study, we found only two trials, which included 34 and 40 eyes, that directly compared the IOP of patients receiving corticosteroids and no injections [41, 44]. Although the authors found that the IOP significantly differed between the two groups, the observed effect was relatively small (the mean difference between the two groups was 3.60 mmHg in one study and 1.30 mmHg in the other; Additional file 1: Table S12) [41, 44]. Our analysis did not show significant results for IOP increase because the effect size was small, and the random-effects models we used in the network meta-analysis generated a wider estimate interval than that for a single study [67]. Therefore, we cannot rule out the risk of IOP elevation after intravitreal corticosteroid injections.

### Limitations

First, we used the mean changes in BCVA instead of the number of patients with improved BCVA (e.g., ETDRS letter change ≥ 10 or 15) as the study outcome. Thus, our study may have excluded studies that only reported the number of patients with improved BCVA. However, we used the mean changes in BCVA because the definition of “improved BCVA” varies among studies. Second, the standard deviation of the mean difference was not reported in some studies. This issue may have induced bias, although we imputed the standard deviation according to Cochrane Handbook guidelines. Third, most included trials used time-domain OCT to measure the CMT, while some trials used different devices. This may also have introduced bias in the CMT assessments. However, we analyzed the CMT change, which may dilute the heterogeneity in OCT devices used because the same device was used for the same patient in individual studies. Fourth, there was only one study with a relatively small sample size investigating the effect of intravitreal TA injections on long-term BCVA. Although our results suggested that multiple injections of TA resulted in the largest improvements in BCVA, the results should be interpreted with caution. TA may play a role in improving long-term outcomes, but the risk for glaucoma and other complications caused by repeated injections should not be ignored and deserves further study [11]. Fifth, the longest follow-up periods varied among different studies, from 3 to 39 months. Our study defined the outcomes as short-term (within 6 months) and long-term (over 1 year). This may have introduced heterogeneity; however, this definition allowed us to include as many studies as possible to make the analysis more meaningful. Last, the short-term change in IOP was only reported in TA studies. The effects of FA or DEX implants on IOP were not assessable by our study. More data are needed to compare the safety of intravitreal FA or DEX implants for the management of DME.

## Conclusions

Intravitreal corticosteroids are effective for treating DME, while the level of efficacy varies across types and dosages of corticosteroids. Intravitreal TA was not inferior to FA or DEX implants regarding both short-term and long-term outcomes, suggesting that TA could be a low-cost option for DME patients requiring corticosteroid treatments. However, the risk of IOP increase should not be ignored. The long-term efficacy and safety of different corticosteroids deserve further study.

## Supplementary Information

**Additional file 1.** Additional Tables, Figures, and Search Strategies.

## Data Availability

The datasets supporting the conclusions of this article are included within the article and its Additional file.

## Abbreviations

VEGF: Vascular endothelial growth factor
TA: Triamcinolone acetonide
FA: Fluocinolone acetonide
DEX: Dexamethasone
PRISMA: Preferred Reporting Items for Systematic Reviews and Meta-Analyses
PRISMA-NMA: Preferred Reporting Items for Systematic Reviews Incorporating Network Meta-Analyses
PROSPERO: Prospectively Registered in the International Prospective Register of Systematic Reviews
BCVA: Best-corrected visual acuity
CMT: Central macular thickness
IOP: Intraocular pressure
ETDRS: Early Treatment Diabetic Retinopathy Study
logMAR: Logarithm of the minimal angle of resolution
CI: Confidence interval
CrI: Credible interval
SUCRA: Surface under the cumulative ranking curve
GRADE: The Grading of Recommendations Assessment, Development, and Evaluation
EURETINA: The European Society of Retina Specialists
