# Supplementary material for: Intravitreal corticosteroids for diabetic macular edema: a network meta-analysis of randomized controlled trials
Source: Eye Vis (Lond). 2021 Oct 11;8:35. doi: 10.1186/s40662-021-00261-3 (PMC8504110; doi:10.1186/s40662-021-00261-3)
Supplement: Supplementary file 1 — Additional file 1. Additional Tables, Figures, and Search Strategies. [file 40662_2021_261_MOESM1_ESM.docx]

**Additional file provided by authors to supplement the manuscript entitled**

**“Intravitreal corticosteroids for diabetic macular edema: a network meta-analysis of randomized controlled trials”**

**Content**

- **Table S1.** Outcome measures extracted or derived from original studies
  [Page 4-7]
- **Table S2.** Risk of bias within individual studies
  [Page 8]
- **Table S3.** Estimates of effects and quality ratings for comparisons of intravitreal corticosteroids to improve short-term BCVA

[Page 9-10]

- **Table S4.** Quality of direct evidence for comparisons of intravitreal corticosteroids to improve short-term BCVA

[Page 11-13]

- **Table S5.** Evaluation of the inconsistency between direct and indirect evidence for the effect of intravitreal corticosteroids on short-term BCVA using node splitting model

[Page 14]

- **Table S6.** Estimates of effects and quality ratings for comparisons of intravitreal corticosteroids to improve long-term BCVA

[Page 15-16]

- **Table S7.** Quality of direct evidence for comparisons of intravitreal corticosteroids to improve long-term BCVA

[Page 17-18]

- **Table S8.** Estimates of effects and quality ratings for comparisons of intravitreal corticosteroids to reduce short-term CMT

[Page 19-21]

- **Table S9.** Quality of direct evidence for comparisons of intravitreal corticosteroids to reduce short-term CMT

[Page 22-23]

- **Table S10.** Evaluation of the inconsistency between direct and indirect evidence for the effect of intravitreal corticosteroids on short-term CMT using node splitting model

[Page 24]

- **Table S11.** Estimates of effects and quality ratings for comparisons of intravitreal corticosteroids for increasing short-term IOP

[Page 25]

- **Table S12.** Quality of direct evidence for comparisons of intravitreal corticosteroids for increasing short-term IOP

[Page 26-27]

- **Table S13.** Evaluation of the inconsistency between direct and indirect evidence for the effect of intravitreal corticosteroids on short-term IOP using node splitting model

[Page 28]

- **Figure S1.** Pooled risk of bias

[Page 29]

- **Figure S2.** Network geometry of different intravitreal corticosteroids to reduce short-term CMT

[Page 30]

- **Figure S3.** Effects of the different intravitreal corticosteroids on short-term CMT

[Page 31]

- **Figure S4.** SUCRA scores for the effects of different intravitreal corticosteroids on short-term CMT

[Page 32]

- **Figure S5.** Network geometry of different intravitreal corticosteroids for increasing short-term IOP

[Page 33]

- **Figure S6.** Effects of the different intravitreal corticosteroids on short-term IOP

[Page 34]

- **eAppendix.** Search strategies

[Page 35-37]

- **References**
  [Page 38-39]

**Table S1. Outcome measures extracted or derived from original studies**

| **First author (year)** | **Intervention category** | **Mean** **BCVA change ± SD** | | **Mean CMT change ± SD** | **Mean IOP change ± SD** |
| --- | --- | --- | --- | --- | --- |
|  |  | **Short-term** | **Long-term** | **Short-term** | **Short-term** |
| Sutter (2004) [1] | TA injection (4–8 mg) | 5.0 ± 1.2  (ETDRS letter, 3-month) | - | –152.0 ± 27.0  (µm, 3-month, measurement device not reported) | - |
|  | Control | –0.1 ± 1.5  (ETDRS letter, 3-month) | - | –36.0 ± 17.0  (µm, 3-month, measurement device not reported) | - |
| Spandau (2005) [2] | TA injection (< 4 mg) | 0.42 ± 2.06*  (Snellen line, 6-month) | - | - | 0.80 ± 1.02*  (mmHg, mean follow-up = 6.6 months) |
|  | TA injection (4–8 mg) | 1.33 ± 2.25*  (Snellen line, 6-month) | - | - | 2.30 ± 0.86*  (mmHg, mean follow-up = 6.6 months) |
|  | TA injection  (≥ 8 mg) | 3.63 ± 4.44*  (Snellen line, 6-month) | - | - | 0.70 ± 1.79*  (mmHg, mean follow-up = 6.6-month) |
| Audren (2006) [3] | TA injection (4–8 mg) | 6.9 ± 10.7  (ETDRS letter, 6-month) | - | –207.9 ± 110.4*  (µm, 6-month, measured based on time-domain OCT) | 0.10 ± 1.8*  (mmHg, 6-month) |
|  | Control | –2.6 ± 7.8  (ETDRS letter, 6-month) | - | –49.8 ± 89.7*  (µm, 6-month, measured based on time-domain OCT) | –1.20 ± 1.52*  (mmHg, 6-month) |
| Audren (2006) [4] | TA injection (<4 mg) | 5.3 ± 7.1  (ETDRS letter, 6-month) | - | –128.2 ± 107.5*  (µm, 6-month, measured based on time-domain OCT) | –1.40 ± 2.41*  (mmHg, 6-month) |
|  | TA injection (4–8 mg) | 5.5 ± 7.6  (ETDRS letter, 6-month) | - | –115.8 ± 87.9*  (µm, 6-month, measured based on time-domain OCT) | –2.30 ± 2.06*  (mmHg, 6-month) |
| Gillies (2006) [5] | TA injection  (4 mg, multiple times) | - | 5.7 ± 11.9  (mean difference between two groups, ETDRS letter, 2-year) | - | - |
|  | Control | - |  | - | - |
| Jonas (2006) [6] | TA injection  (≥ 8 mg) | –0.33 ± 0.24  (logMAR, mean follow-up = 10 months) | - | - | 2.20 ± 3.92*  (mmHg, mean follow-up = 10 months) |
|  | Control | –0.05 ± 0.36  (LogMAR, mean follow-up = 10 months) | - | - | –1.40 ± 2.42*  (mmHg, mean follow-up = 10 months) |
| Lam (2007) [7] | TA injection (4–8 mg) | 3.1 ± 10.0  (ETDRS letters, 26-week) | - | 28.7 ± 49.1*  (µm, 26-week, measured based on time-domain OCT) | - |
|  | TA injection  (≥ 8 mg) | 9.9± 11.7  (ETDRS letters, 26-week) | - | 60.5 ± 41.1*  (µm, 26-week, measured based on time-domain OCT) | - |
| Dehghan (2008) [8] | TA injection (4–8 mg) | –0.04 ± 0.04  (logMAR, 4-month) | - | –31.0 ± 90.6*  (µm, 4-month, measured based on time-domain OCT) | - |
|  | Control | 0.07 ± 0.04  (logMAR, 4-month) | - | 12.0 ± 103.2*  (µm, 4-month, measured based on time-domain OCT) | - |
| Hauser (2008) [9] | TA injection (< 4 mg) | 8.1 ± 10.6  (ETDRS letter, 6-month) | - | –63.0 ± 90.0  (µm, 6-month, measured based on RTA) | 0.50 ± 2.46*  (mmHg, 6-month) |
|  | TA injection (4–8 mg) | 4.6 ± 7.6  (ETDRS letter, 6-month) | - | –9.0 ±136.0  (µm, 6-month, measured based on RTA) | 2.00 ± 4.83*  (mmHg, 6-month) |
| Kim (2008) [10] | TA injection (< 4 mg) | 3.7 ± 5.0  (ETDRS letter, 6-month) | - | - | - |
|  | TA injection (4–8 mg) | 7.4 ± 6.9  (ETDRS letter, 6-month) | - | - | - |
| Larsson (2009) [11] | TA injection (4–8 mg) | 5.0 ± 4.4  (ETDRS letter, 3-month) | - | - | - |
|  | Control | –1.7 ± 7.5  (ETDRS letter, 3-month) | - | - | - |
| Campochiaro (2010) [12] | FA implant (0.2 µg/d) | 2.7 ± 2.5  (ETDRS letter, 6-month) | 1.3 ± 2.6  (ETDRS letter, 1- year) | –87.0 ± 98.9  (µm, 6-month, measured based on time-domain OCT) | - |
|  | FA implant (0.5 µg/d) | 6.9 ± 3.1  (ETDRS letter, 6-month) | 5.7 ± 3.2  (ETDRS letter, 1-year) | 175.7 ± 49.1  (µm, 6-month, measured based on time-domain OCT) | - |
| Chan (2010) [13] | DEX injection (400 µg) | 0.14 ± 0.13  (LogMAR, 13-week) | - | - | - |
|  | DEX injection (800 µg) | –0.09 ± 0.22  (LogMAR, 13-week) | - | - | - |
| Campochiaro (2011, 2012) [14, 15] | FA implant (0.2 µg/d) | 1.6± 5.2†  (ETDRS letter, 6-month) | 5.3 ± 8.4†  (ETDRS letter, 3-year) | –140.0‡  (µm, 6-month, measured based on time-domain OCT) | - |
|  | FA implant (0.5 µg/d) | 6.2 ± 6.4†  (ETDRS letter, 6-month) | 5.3 ± 8.6†  (ETDRS letter, 3-year) | –168.0‡  (µm, 6-month, measured based on time-domain OCT) | - |
|  | Control | 5.8 ± 6.1†  (ETDRS letter, 6-month) | 2.0 ± 7.6†  (ETDRS letter, 3-year) | –55.0‡  (µm, 6-month, measured based on time-domain OCT) | - |
| Pearson (2011) [16] | FA implant (0.5 µg/d) | - | - | –148.0‡  (µm, 6-month, measurement device not reported) | - |
|  | Control | - | - | 22.0‡  (µm, 6-month, measurement device not reported) | - |
| Boyer (2014) [17] and Danis (2016) [18] | DEX implant (350 µg) | 3.0 ± 5.2†  (ETDRS letter, 6-month) | 2.6 ± 6.2†  (ETDRS letter, 3-year) | –50.0‡  (µm, 6-month, measured based on time-domain OCT) | - |
|  | DEX implant (700 µg) | 4.1 ± 4.3†  (ETDRS letter, 6-month) | 2.2 ± 6.7†  (ETDRS letter, 3-year) | –63.0‡  (µm, 6-month, measured based on time-domain OCT) | - |
|  | Control | 1.5 ± 4.8†  (ETDRS letter, 6-month) | 0.4 ± 5.7†  (ETDRS letter, 3-year) | –25.0‡  (µm, 6-month, measured based on time-domain OCT) | - |
| Lodhi (2015) [19] | TA injection (< 4 mg) | –0.44 ± 0.33*  (logMAR, 6-month) | - | - | 0.60 ± 1.59*  (mmHg, 6-month) |
|  | TA injection (4–8 mg) | –0.37 ± 0.33*  (logMAR, 6-month) | - | - | 1.00 ± 1.34*  (mmHg, 6-month) |
| Mylonas (2016) [20] | TA injection (4–8 mg) | 8.0 ± 8.2*  (ETDRS letter, 6-month) | - | –151.0 ± 76.1*  (µm, 6-month, measured based on spectral-domain OCT) | - |
|  | DEX implant (700 µg) | 6.0 ± 7.8*  (ETDRS letter, 6-month) | - | –44.0 ± 96.9*  (µm, 6-month, measured based on spectral-domain OCT) | - |
| Zhou (2016) [21] | TA injection (< 4 mg) | 0.11 ± 0.10*  (Snellen line, 6-month) | - | –259.4 ± 116.2*  (µm, 6-month, measurement device not reported) | - |
|  | TA injection (4–8 mg) | 0.17 ± 0.08*  (Snellen line, 6-month) | - | –285.6 ± 115.6*  (µm, 6-month, measurement device not reported) | - |

*SD* standard deviation; *BCVA* best-corrected visual acuity; *CMT* central macular thickness; *IOP* intraocular pressure; *TA* triamcinolone acetonide; *ETDRS* Early Treatment Diabetic Retinopathy Study; *OCT* optical coherence tomography; *logMAR* logarithm of the minimum angle of resolution; *RTA* retinal thickness analyzer; *FA* fluocinolone acetonide; *DEX* dexamethasone

* The mean and standard deviation were calculated using the method described by Cochrane Handbook for Systematic Reviews of Interventions (Version 5.1.0, Part 3, Chapter 16.1.3.2) [22]

† The mean and standard deviation were estimated from the plots in original studies

‡ The standard deviation was not calculatable based on the data reported by original studies. We estimated their standard deviations by referring to other studies during meta-analysis

**Table S2. Risk of bias within individual studies**

| **Study** | **Selection bias** | | **Performance bias*** | **Detection bias** | **Attrition bias** | **Reporting bias** | **Other**  **bias** | **Overall bias** |
| --- | --- | --- | --- | --- | --- | --- | --- | --- |
| **First author, year** | **Random sequence generation** | **Allocation concealment** | **Blinding of participants and personnel** | **Blinding of outcome assessment** | **Incomplete outcome data assessments** | **Selective reporting** |  |  |
| Sutter (2004) [1] | **+** | **+** | **+** | **+** | **+** | **+** | **+** | **+** |
| Spandau (2005) [2] | **+** | **+** | **+** | **+** | **+** | **+** | **+** | **+** |
| Audren (2006) [3] | **+** | **+** | **+** | **+** | **+** | **+** | **+** | **+** |
| Audren (2006) [4] | **+** | **+** | **+** | **+** | **+** | **+** | **+** | **+** |
| Gillies (2006) [5] | **+** | **+** | **+** | **+** | **+** | **+** | **+** | **+** |
| Jonas (2006) [6] | **+** | **+** | **+** | **+** | **+** | **+** | **+** | **+** |
| Lam (2007) [7] | **+** | **+** | **+** | **+** | **+** | **?** | **+** | **?** |
| Dehghan (2008) [8] | **+** | **+** | **+** | **+** | **+** | **+** | **+** | **+** |
| Hauser (2008) [9] | **+** | **+** | **?** | **+** | **+** | **+** | **+** | **?** |
| Kim (2008) [10] | **+** | **+** | **+** | **+** | **+** | **+** | **+** | **+** |
| Larsson (2009) [11] | **+** | **+** | **+** | **+** | **+** | **+** | **+** | **+** |
| Campochiaro (2010) [12] | **+** | **+** | **+** | **+** | **+** | **+** | **+** | **+** |
| Chan (2010) [13] | **+** | **+** | **+** | **+** | **+** | **+** | **+** | **+** |
| Campochiaro (2011) [14] | **+** | **+** | **+** | **+** | **+** | **+** | **+** | **+** |
| Pearson (2011) [16] | **+** | – | – | **+** | **+** | **+** | **+** | – |
| Campochiaro (2012) [15] | **+** | **+** | **+** | **+** | **+** | **+** | **+** | **+** |
| Boyer (2014) [17] | **+** | **+** | **+** | **+** | **+** | **+** | **+** | **+** |
| Lodhi (2015) [19] | **+** | **?** | **?** | **+** | **+** | **+** | **+** | **?** |
| Danis (2016) [18] | **+** | **+** | **+** | **+** | **+** | **+** | **+** | **+** |
| Mylonas (2016) [20] | **+** | **+** | – | **?** | **+** | **+** | **+** | – |
| Zhou (2016) [21] | **+** | **+** | **?** | **?** | **+** | **+** | **+** | **?** |

Low (+), low risk of bias; High (–), high risk of bias; Unclear (?), unclear risk of bias according to the relative information.

**Table S3. Estimates of effects and quality ratings for comparisons of intravitreal corticosteroids to improve short-term BCVA**

| **Comparison** | **Direct evidence*** | | **Indirect evidence†** | | **Network meta-analysis‡** | |
| --- | --- | --- | --- | --- | --- | --- |
|  | **Mean difference of BCVA (logMAR) change between two groups [95% CI]** | **Quality** | **Mean difference of BCVA (logMAR) change between two groups [95% CrI]** | **Quality** | **Mean difference of BCVA (logMAR) change between two groups [95% CrI]** | **Quality** |
| TA injection (≥ 8 mg) *vs.* Control | –0.28 [–0.50, –0.06] | Very low | –0.27 [–0.42, –0.13] | Low | –0.27 [–0.40, –0.15] | Low |
| TA injection (4–8 mg) *vs.* Control | –0.11 [–0.12, –0.10] | High | –0.10 [–0.26, 0.06] | Very low | –0.12 [–0.18, –0.06] | High |
| TA injection (< 4 mg) *vs.* Control | - | - | –0.07 [–0.16, 0.01] | Low | –0.07 [–0.16, 0.01] | Low |
| FA implant (0.5 µg/d) *vs.* Control | –0.08 [–0.10, –0.06] | Moderate | - | - | –0.10 [–0.21, –0.01] | Moderate |
| FA implant (0.2 µg/d) *vs.* Control | –0.09 [–0.11, –0.07] | Moderate | - | - | –0.07 [–0.17, 0.03] | Moderate |
| DEX implant (700 µg) *vs.* Control | –0.05 [–0.07, –0.04] | Moderate | –0.08 [–0.20, 0.04] | Moderate | –0.06 [–0.11, –0.01] | Moderate |
| DEX implant (350 µg) *vs.* Control | –0.03 [–0.04, –0.02] | Moderate | - | Moderate | –0.03 [–0.10, 0.03] | Moderate |
| TA injection (≥ 8 mg) *vs.*  TA injection (4–8 mg) | –0.14 [–0.26, –0.02] | Low | –0.16 [–0.41, 0.09] | Very low | –0.16 [–0.27, –0.04] | Low |
| TA injection (≥ 8 mg) *vs.*  TA injection (< 4 mg) | –0.41 [–0.74, –0.08] | Very low | –0.18 [–0.33, –0.02] | Low | –0.20 [–0.34, –0.07] | Low |
| TA injection (≥ 8 mg) *vs.*  FA implant (0.5 µg/d) | - | - | –0.17 [–0.33, –0.01] | Very low | –0.17 [–0.33, –0.01] | Very low |
| TA injection (≥ 8 mg) *vs.*  FA implant (0.2 µg/d) | - | - | –0.20 [–0.37, –0.05] | Very low | –0.20 [–0.37, –0.05] | Very low |
| TA injection (≥ 8 mg) *vs.*  DEX implant (700 µg) | - | - | –0.21 [–0.36, –0.07] | Very low | –0.21 [–0.36, –0.07] | Very low |
| TA injection (≥ 8 mg) *vs.*  DEX implant (350 µg) | - | - | –0.24 [–0.40, –0.09] | Very low | –0.24 [–0.40, –0.09] | Very low |
| TA injection (4–8 mg) *vs.* TA injection (< 4 mg) | –0.04 [–0.11, 0.03] | Low | - | - | –0.05 [–0.11, 0.02] | Low |
| TA injection (4–8 mg) *vs.*  FA implant (0.5 µg/d) | - | - | –0.01 [–0.13, 0.10] | Moderate | –0.01 [–0.13, 0.10] | Moderate |
| TA injection (4–8 mg) *vs.*  FA implant (0.2 µg/d) | - | - | –0.05 [–0.17, 0.06] | Moderate | –0.05 [–0.17, 0.06] | Moderate |
| TA injection (4–8 mg) *vs.*  DEX implant (700 µg) | –0.04 [–0.16, 0.08] | Very low | –0.07 [–0.21, 0.06] | Moderate | –0.06 [–0.15, 0.04] | Moderate |
| TA injection (4–8 mg) *vs.*  DEX implant (350 µg) | - | - | –0.08 [–0.20, 0.03] | Low | –0.08 [–0.20, 0.03] | Low |
| TA injection (< 4 mg) *vs.*  FA implant (0.5 µg/d) | - | - | 0.03 [–0.10, 0.16] | Low | 0.03 [–0.10, 0.16] | Low |
| TA injection (< 4 mg) *vs.*  FA implant (0.2 µg/d) | - | - | 0.00 [–0.14, 0.12] | Low | 0.00 [–0.14, 0.12] | Low |
| TA injection (< 4 mg) *vs.*  DEX implant (700 µg) | - | - | –0.01 [–0.13, 0.10] | Low | –0.01 [–0.13, 0.10] | Low |
| TA injection (< 4 mg) *vs.*  DEX implant (350 µg) | - | - | –0.04 [–0.17, 0.09] | Low | –0.04 [–0.17, 0.09] | Low |
| FA implant (0.5 µg/d) *vs.*  FA implant (0.2 µg/d) | –0.04 [–0.13, 0.05] | Moderate | - | - | –0.03 [–0.11, 0.04] | Moderate |
| FA implant (0.5 µg/d) *vs.*  DEX implant (700 µg) | - | - | –0.05 [–0.18, 0.09] | Moderate | –0.05 [–0.18, 0.09] | Moderate |
| FA implant (0.5 µg/d) *vs.*  DEX implant (350 µg) | - | - | –0.07 [–0.21, 0.08] | Moderate | –0.07 [–0.21, 0.08] | Moderate |
| FA implant (0.2 µg/d) *vs.*  DEX implant (700 µg) | - | - | –0.01 [–0.14, 0.13] | Moderate | –0.01 [–0.14, 0.13] | Moderate |
| FA implant (0.2 µg/d) *vs.*  DEX implant (350 µg) | - | - | –0.04 [–0.18, 0.11] | Moderate | –0.04 [–0.18, 0.11] | Moderate |
| DEX implant (700 µg) *vs.*  DEX implant (350 µg) | –0.02 [–0.04, –0.01] | Moderate | - | - | –0.03 [–0.13, 0.07] | Moderate |

*BCVA* best-corrected visual acuity; *logMAR* logarithm of the minimum angle of resolution; *CI* confident interval; *CrI* credible interval; *TA* triamcinolone acetonide; *FA* fluocinolone acetonide; *DEX* dexamethasone; *GRADE* Grading of Recommendations Assessment, Development, and Evaluation

* Direct evidence was based on pairwise meta-analysis which aggregates the results of the head-to-head comparisons between different treatments. Quality of direct evidence was assessed per the GRADE guidelines [23], with details of assessment presented in Table S4 in Additional files

† Indirect evidence was based on network meta-analysis which estimates the difference between two treatments based on their comparisons with other treatments in the network, but not their direct comparisons. Quality of indirect evidence was assessed per the GRADE guidelines for network meta-analysis [24]

‡ The quality of each network estimate was assessed per the GRADE guidelines for network meta-analysis [24]; it was determined by the quality of direct or indirect evidence whichever was higher

**Table S4. Quality of direct evidence for comparisons of intravitreal corticosteroids to improve short-term BCVA**

| **Comparison** | **Number of studies, (sample size)** | **Required information size met*** | **Mean difference of BCVA (LogMAR) change between two groups [95% CI]** | **Overall ROB (high and unclear)** | **I^2^** | **Egger’s test**  **(P value)** | **Quality of evidence** | **Comments** |
| --- | --- | --- | --- | --- | --- | --- | --- | --- |
| TA injection (≥ 8 mg) vs. Control | 1 (40)[6] | N/A | –0.28 [–0.50, –0.06] | 0% | N/A | N/A | Very low | Result was based on a randomized controlled trial with small sample size |
| TA injection (4–8 mg) vs. Control | 4 (210)[1, 3, 8, 11] | Yes | –0.11 [–0.12, –0.10] | 0% | 0 | 0.13 | High | - Imprecision ☒  - Indirectness ☒  - Inconsistency ☒  - Study limitations (ROB) ☒  - Publication bias ☒ |
| FA implant (0.5 µg/d) vs. Control | 1 (578)[14] | N/A | –0.08 [–0.10, –0.06] | 0% | N/A | N/A | Moderate | Result was based on a randomized controlled trial with a large sample size |
| FA implant (0.2 µg/d) vs. Control | 1 (560)[14] | N/A | –0.09 [–0.11, –0.07] | 0% | N/A | N/A | Moderate | Result was based on a randomized controlled trial with a large sample size |
| DEX implant (700 µg) *vs.* Control | 1 (701)[17] | N/A | –0.05 [–0.07, –0.04] | 0% | N/A | N/A | Moderate | Result was based on a randomized controlled trial with a large sample size |
| DEX implant (350 µg) *vs.* Control | 1 (697)[17] | N/A | –0.03 [–0.04, –0.02] | 0% | N/A | N/A | Moderate | Result was based on a randomized controlled trial with a large sample size |
| TA injection (≥ 8 mg) *vs.*  TA injection (4–8 mg) | 2 (62)[2, 7] | No | –0.14 [–0.26, –0.02] | 50% | 0 | N/A | Low | - Imprecision ☐†  - Indirectness ☒  - Inconsistency ☒  - Study limitations (ROB) ☐‡  - Publication bias ☒ |
| TA injection (≥ 8 mg) *vs.*  TA injection (< 4 mg) | 1 (17)[2] | N/A | –0.41 [–0.74, –0.08] | 0% | N/A | N/A | Very low | Result was based on a randomized controlled trial with a small sample size |
| TA injection (4–8 mg) *vs.* TA injection (< 4 mg) | 6 (202)[2, 4, 9, 10, 19, 21] | Yes | –0.04 [–0.11, 0.03] | 50% | 55 | 0.84 | Low | - Imprecision ☒  - Indirectness ☒  - Inconsistency ☐§  - Study limitations (ROB) ☐‡  - Publication bias ☒ |
| TA injection (4–8 mg) vs.  DEX implant (700 µg) | 1 (29)[20] | N/A | –0.04 [–0.16, 0.08] | 100% | N/A | N/A | Very low | Result was based on a randomized controlled trial with a high risk of bias |
| FA implant (0.5 µg/d) vs.  FA implant (0.2 µg/d) | 2 (805)[12, 14] | Yes | –0.04 [–0.13, 0.05] | 0% | 95 | N/A | Moderate | - Imprecision ☒  - Indirectness ☒  - Inconsistency ☐§  - Study limitations (ROB) ☒  - Publication bias ☒ |
| DEX implant (700 µg) vs.  DEX implant (350 µg) | 1 (698)[17] | N/A | –0.02 [–0.04, –0.01] | 0% | N/A | N/A | Moderate | Result was based on a randomized controlled trial with a large sample size |

The mean difference and 95% CI were calculated using pairwise meta-analysis. The actual sample size is the sum of patients included in the body of evidence. The evidence quality was graded according to the Grading of Recommendations Assessment, Development, and Evaluation (GRADE) guidelines [23].

*BCVA* best-corrected visual acuity; *logMAR* logarithm of the minimum angle of resolution; *CI* confident interval; *ROB* risk of bias; *TA* triamcinolone acetonide; *N/A* not applicable; *FA* fluocinolone acetonide; *DEX* dexamethasone

* Required information size was evaluated according to GRADE guidelines 6 [25]. When calculating the required information size, we chose an α error of 0.05, β error of 0.2, a mean difference of 0.20 (implying a judgment that reductions in logMAR value of more than 0.2 are important), and a standard deviation of 0.32 (according to four relevant studies).[1, 2, 14, 17] It yields the corresponding required information size of 84

† Downgraded one level for imprecision because the required information size was not met [25]

‡ Downgraded one level for high overall risk of bias within studies if the percentage of the studies with a high or unclear overall risk of bias within the body of evidence was ≥ 50% [22]. The overall risk of bias within individual studies was based on the assessment of the risks of bias for different domains (Table S2) [26]

§ Downgraded one level for inconsistency as a result of high statistical heterogeneity (I^2^ ≥ 40%) that could not be explained by subgroup analyses [27]

☒ indicated no downgrading

☐ indicated that the quality of evidence was downgraded for the factor

**Table S5. Evaluation of the inconsistency between direct and indirect evidence for the effect of intravitreal corticosteroids on short-term BCVA using node splitting model [28]**

| **Comparison** | **Direct evidence** | **Indirect evidence** | ***P* value** |
| --- | --- | --- | --- |
|  | **Mean difference of BCVA (logMAR) change between two groups [95% CrI]** | |  |
| TA injection (≥ 8 mg) *vs.* Control | –0.28 [–0.53, –0.04] | –0.27 [–0.42, –0.13] | 0.96 |
| TA injection (4–8 mg) *vs.* Control | –0.12 [–0.19, –0.06] | –0.10 [–0.26, 0.06] | 0.81 |
| DEX implant (700 µg) *vs.* Control | –0.05 [–0.11, 0.01] | –0.08 [–0.20, 0.04] | 0.77 |
| TA injection (≥ 8 mg) *vs.*  TA injection (4–8 mg) | –0.17 [–0.26, –0.07] | –0.16 [–0.41, 0.09] | 0.98 |
| TA injection (≥ 8 mg) *vs.*  TA injection (< 4 mg) | –0.41 [–0.76, –0.06] | –0.18 [–0.33, –0.02] | 0.24 |
| TA injection (4–8 mg) *vs.*  DEX implant (700 µg) | –0.04 [–0.17, 0.08] | –0.07 [–0.21, 0.06] | 0.76 |

*BCVA* best-corrected visual acuity; *logMAR* logarithm of the minimum angle of resolution; *CrI* credible interval; *TA* triamcinolone acetonide; *DEX*, dexamethasone

**Table S6. Estimates of effects and quality ratings for comparisons of intravitreal corticosteroids to improve long-term BCVA**

| **Comparison** | **Direct evidence*** | | **Indirect evidence†** | | **Network meta-analysis‡** | |
| --- | --- | --- | --- | --- | --- | --- |
|  | **Mean difference of BCVA (logMAR) change between two groups [95% CI]** | **Quality** | **Mean difference of BCVA (logMAR) change between two groups [95% CrI]** | **Quality** | **Mean difference of BCVA (LogMAR) change between two groups [95% CrI]** | **Quality** |
| TA injection (4 mg, multiple times) *vs.* Control | –0.11 [–0.20, –0.03] | Low | - | - | –0.11 [–0.21, –0.02] | Low |
| FA implant (0.5 µg/d) *vs.* Control | –0.07 [–0.09, –0.04] | Moderate | - | - | –0.09 [–0.15, –0.03] | Moderate |
| FA implant (0.2 µg/d) *vs.* Control | –0.07 [–0.09, –0.04] | Moderate | - | - | –0.09 [–0.14, –0.02] | Moderate |
| DEX implant (700 µg) *vs.* Control | –0.04 [–0.05, –0.02] | Moderate | - | - | –0.04 [–0.10, 0.01] | Moderate |
| DEX implant (350 µg) *vs.* Control | –0.04 [–0.06, –0.03] | Moderate | - | - | –0.04 [–0.07, –0.01] | Moderate |
| TA injection (4 mg, multiple times) *vs.* FA implant (0.5 µg/d) | - | - | –0.03 [–0.15, 0.12] | Low | –0.03 [–0.15, 0.12] | Low |
| TA injection (4 mg, multiple times) *vs.* FA implant (0.2 µg/d) | - | - | –0.07 [–0.25, 0.11] | Low | –0.07 [–0.25, 0.11] | Low |
| TA injection (4 mg, multiple times) *vs.* DEX implant (700 µg) | - | - | –0.08 [–0.31, 0.15] | Low | –0.08 [–0.31, 0.15] | Low |
| TA injection (4 mg, multiple times) *vs.* DEX implant (350 µg) | - | - | –0.07 [–0.30, 0.16] | Low | –0.07 [–0.30, 0.16] | Low |
| FA implant (0.5 µg/d) *vs.*  FA implant (0.2 µg/d) | –0.04 [–0.13, 0.04] | Moderate | - | - | –0.04 [–0.16, 0.07] | Moderate |
| FA implant (0.5 µg/d) *vs.*  DEX implant (700 µg) | - | - | –0.05 [–0.26, 0.16] | Moderate | –0.05 [–0.26, 0.16] | Moderate |
| FA implant (0.5 µg/d) *vs.*  DEX implant (350 µg) | - | - | –0.04 [–0.26, 0.17] | Moderate | –0.04 [–0.26, 0.17] | Moderate |
| FA implant (0.2 µg/d) *vs.*  DEX implant (700 µg) | - | - | –0.01 [–0.22, 0.20] | Moderate | –0.01 [–0.22, 0.20] | Moderate |
| FA implant (0.2 µg/d) *vs.*  DEX implant (350 µg) | - | - | 0.00 [–0.21, 0.21] | Moderate | 0.00 [–0.21, 0.21] | Moderate |
| DEX implant (700 µg) *vs.*  DEX implant (350 µg) | 0.01 [–0.01, 0.03] | Moderate | - | - | 0.01 [–0.14, 0.16] | Moderate |

*BCVA* best-corrected visual acuity; *logMAR* logarithm of the minimum angle of resolution; *CI* confident interval; *CrI* credible interval; *TA* triamcinolone acetonide; *FA* fluocinolone acetonide; *DEX* dexamethasone; *GRADE* Grading of Recommendations Assessment, Development, and Evaluation

* Direct evidence was based on pairwise meta-analysis which aggregates the results of the head-to-head comparisons between different treatments. Quality of direct evidence was assessed per the GRADE guidelines [23], with details of assessment presented in Table S7 in Additional files

† Indirect evidence was based on network meta-analysis which estimates the difference between two treatments based on their comparisons with other treatments in the network, but not their direct comparisons. Quality of indirect evidence was assessed per the GRADE guidelines for network meta-analysis [24]

‡ The quality of each network estimate was assessed per the GRADE guidelines for network meta-analysis [24]; it was determined by the quality of direct or indirect evidence whichever was higher

**Table S7. Quality of direct evidence for comparisons of intravitreal corticosteroids to improve long-term BCVA**

| **Comparison** | **Number of studies, (sample size)** | **Required information size met*** | **Mean difference of BCVA (logMAR) change between two groups [95% CI]** | **Overall ROB (high and unclear)** | **I^2^** | **Egger’s test**  **(*P* value)** | **Quality of evidence** | **Comments** |
| --- | --- | --- | --- | --- | --- | --- | --- | --- |
| TA injection (4 mg, multiple times) vs. Control | 1 (60) [6] | N/A | –0.11 [–0.20, –0.03] | 0% | N/A | N/A | Low | Result was based on a randomized controlled trial |
| FA implant (0.5 µg/d) vs. Control | 1 (580) [15] | N/A | –0.07 [–0.09, –0.04] | 0% | N/A | N/A | Moderate | Result was based on a randomized controlled trial with a large sample size |
| FA implant (0.2 µg/d) vs. Control | 1 (561) [15] | N/A | –0.07 [–0.09, –0.04] | 0% | N/A | N/A | Moderate | Result was based on a randomized controlled trial with a large sample size |
| DEX implant (700 µg) *vs.* Control | 1 (690) [18] | N/A | –0.04 [–0.05, –0.02] | 0% | N/A | N/A | Moderate | Result was based on a randomized controlled trial with a large sample size |
| DEX implant (350 µg) *vs.* Control | 1 (686) [18] | N/A | –0.04 [–0.06, –0.03] | 0% | N/A | N/A | Moderate | Result was based on a randomized controlled trial with a large sample size |
| FA implant (0.5 µg/d) vs.  FA implant (0.2 µg/d) | 2 (808) [12, 15] | Yes | –0.04 [–0.13, 0.04] | 0% | 93 | N/A | Moderate | - Imprecision ☒  - Indirectness ☒  - Inconsistency ☐†  - Study limitations (ROB) ☒  - Publication bias ☒ |
| DEX implant (700 µg) vs.  DEX implant (350 µg) | 1 (692) [18] | N/A | 0.01 [–0.01, 0.03] | 0% | N/A | N/A | Moderate | Result was based on a randomized controlled trial with a large sample size |

The mean difference and 95% CI were calculated using pairwise meta-analysis. The actual sample size is the sum of patients included in the body of evidence. The evidence quality was graded according to the Grading of Recommendations Assessment, Development, and Evaluation (GRADE) guidelines [23].

*BCVA* best-corrected visual acuity; *logMAR* logarithm of the minimum angle of resolution; *CI* confident interval; *ROB* risk of bias; *TA* triamcinolone acetonide; *N/A* not applicable; *FA* fluocinolone acetonide; *DEX* dexamethasone

* Required information size was evaluated according to GRADE guidelines 6 [25]. When calculating the required information size, we chose an α error of 0.05, β error of 0.2, a mean difference of 0.20 (implying a judgment that reductions in logMAR value of more than 0.2 are important), and a standard deviation of 0.32 (according to four relevant studies) [1, 2, 14, 17]. It yields the corresponding required information size of 84

† Downgraded one level for inconsistency as a result of high statistical heterogeneity (I^2^ ≥ 40%) that could not be explained by subgroup analyses [27].

☒ indicated no downgrading

☐ indicated that the quality of evidence was downgraded for the factor

**Table S8. Estimates of effects and quality ratings for comparisons of intravitreal corticosteroids to reduce short-term CMT**

| **Comparison** | **Direct evidence*** | | **Indirect evidence†** | | **Network meta-analysis‡** | |
| --- | --- | --- | --- | --- | --- | --- |
|  | **Mean difference of CMT (µm) change between two groups [95% CI]** | **Quality** | **Mean difference of CMT (µm) change between two groups [95% CrI]** | **Quality** | **Mean difference of CMT (µm) change between two groups [95% CrI]** | **Quality** |
| TA injection (≥ 8 mg) *vs.* Control | - | - | –187.0 [–308.0, –60.0] | Very low | –187.0 [–308.0, –60.0] | Very low |
| TA injection (4–8 mg) *vs.* Control | –102.5 [–157.3, –47.7] | Moderate | –130.0 [–310.0, 44.0] | Very low | –106.5 [–164.4, –50.2] | Moderate |
| TA injection (< 4 mg) *vs.* Control | - | - | –115.0 [–207.4, –27.2] | Moderate | –115.0 [–207.4, –27.2] | Moderate |
| FA implant (0.5 µg/d) *vs.* Control | –134.1 [–188.0, –80.1] | Low | - | - | –140.0 [–217.1, –72.1] | Low |
| FA implant (0.2 µg/d) *vs.* Control | –85.0 [–100.8, –69.2] | Moderate | –82.0 [–210.0, 47.0] | Low | –84.4 [–137.3, –30.9] | Moderate |
| DEX implant (700 µg) *vs.* Control | –25.0 [–38.3, –11.7] | Moderate | 5.1 [–100.0, 110.0] | Very low | –26.1 [–43.8, –6.4] | Moderate |
| DEX implant (350 µg) *vs.* Control | –38.0 [–51.3, –24.7] | Moderate | - | - | –33.1 [–57.7, –13.7] | Moderate |
| TA injection (≥ 8 mg) *vs.*  TA injection (4–8 mg) | –80.3 [–123.0, –37.6] | Very low | - | - | –80.3 [–187.3, 26.4] | Very low |
| TA injection (≥ 8 mg) *vs.*  TA injection (< 4 mg) | - | - | –71.7 [–196.4, 56.2] | Very low | –71.7 [–196.4, 56.2] | Very low |
| TA injection (≥ 8 mg) *vs.*  FA implant (0.5 µg/d) | - | - | –46.6 [–184.3, 96.0] | Very low | –46.6 [–184.3, 96.0] | Very low |
| TA injection (≥ 8 mg) *vs.*  FA implant (0.2 µg/d) | - | - | –102.4 [–251.1, 42.7] | Very low | –102.4 [–251.1, 42.7] | Very low |
| TA injection (≥ 8 mg) *vs.*  DEX implant (700 µg) | - | - | –170.5 [–307.1, –38.8] | Very low | –170.5 [–307.1, –38.8] | Very low |
| TA injection (≥ 8 mg) *vs.*  DEX implant (350 µg) | - | - | –153.9 [–305.6, –4.3] | Very low | –153.9 [–305.6, –4.3] | Very low |
| TA injection (4–8 mg) *vs.* TA injection (< 4 mg) | 5.4 [–37.6, 48.3] | Moderate | - | - | 8.7 [–59.5, 78.7] | Moderate |
| TA injection (4–8 mg) *vs.*  FA implant (0.5 µg/d) | - | - | 33.7 [–56.0, 129.0] | Low | 33.7 [–56.0, 129.0] | Low |
| TA injection (4–8 mg) *vs.*  FA implant (0.2 µg/d) | - | - | –21.9 [–126.0, 76.6] | Moderate | –21.9 [–126.0, 76.6] | Moderate |
| TA injection (4–8 mg) *vs.*  DEX implant (700 µg) | –107.0 [–170.2, –43.8] | Very low | –78.0 [–210.0, 55.0] | Moderate | –90.3 [–175.3, –11.1] | Moderate |
| TA injection (4–8 mg) *vs.*  DEX implant (350 µg) | - | - | –72.9 [–181.1, 28.9] | Moderate | –72.9 [–181.1, 28.9] | Moderate |
| TA injection (< 4 mg) *vs.*  FA implant (0.5 µg/d) | - | - | 25.2 [–89.3, 141.6] | Low | 25.2 [–89.3, 141.6] | Low |
| TA injection (< 4 mg) *vs.*  FA implant (0.2 µg/d) | - | - | –30.5 [–157.3, 87.5] | Moderate | –30.5 [–157.3, 87.5] | Moderate |
| TA injection (< 4 mg) *vs.*  DEX implant (700 µg) | - | - | –99.0 [–210.7, 5.0] | Very low | –99.0 [–210.7, 5.0] | Very low |
| TA injection (< 4 mg) *vs.*  DEX implant (350 µg) | - | - | –81.7 [–211.5, 38.4] | Moderate | –81.7 [–211.5, 38.4] | Moderate |
| FA implant (0.5 µg/d) *vs.*  FA implant (0.2 µg/d) | –53.5 [–112.2, 5.2] | Moderate | - | - | –55.7 [–131.7, 10.0] | Moderate |
| FA implant (0.5 µg/d) *vs.*  DEX implant (700 µg) | - | - | –123.6 [–236.3, –23.3] | Low | –123.6 [–236.3, –23.3] | Low |
| FA implant (0.5 µg/d) *vs.*  DEX implant (350 µg) | - | - | –106.8 [–232.4, 6.2] | Low | –106.8 [–232.4, 6.2] | Low |
| FA implant (0.2 µg/d) *vs.*  DEX implant (700 µg) | - | - | –68.0 [–184.7, 46.1] | Moderate | –68.0 [–184.7, 46.1] | Moderate |
| FA implant (0.2 µg/d) *vs.*  DEX implant (350 µg) | - | - | –51.3 [–178.5, 75.1] | Moderate | –51.3 [–178.5, 75.1] | Moderate |
| DEX implant (700 µg) *vs.*  DEX implant (350 µg) | 13.0 [–0.3, 26.3] | Moderate | - | - | 16.7 [–75.5, 111.8] | Moderate |

*CMT* central macular thickness; *CI* confident interval; *CrI* credible interval; *TA* triamcinolone acetonide; *FA* fluocinolone acetonide; *DEX* dexamethasone; *GRADE* Grading of Recommendations Assessment, Development, and Evaluation

* Direct evidence was based on pairwise meta-analysis which aggregates the results of the head-to-head comparisons between different treatments. Quality of direct evidence was assessed per the GRADE guidelines [23], with details of assessment presented in Table S9 in Additional files

† Indirect evidence was based on network meta-analysis which estimates the difference between two treatments based on their comparisons with other treatments in the network, but not their direct comparisons. Quality of indirect evidence was assessed per the GRADE guidelines for network meta-analysis [24]

‡ The quality of each network estimate was assessed per the GRADE guidelines for network meta-analysis [24]; it was determined by the quality of direct or indirect evidence whichever was higher

**Table S9. Quality of direct evidence for comparisons of intravitreal corticosteroids to reduce short-term CMT**

| **Comparison** | **Number of studies, (sample size)** | **Required information size met*** | **Mean difference of CMT (µm) change between two groups [95% CI]** | **Overall ROB (high and unclear)** | **I^2^** | **Egger’s test**  **(*P* value)** | **Quality of evidence** | **Comments** |
| --- | --- | --- | --- | --- | --- | --- | --- | --- |
| TA injection (4–8 mg) vs. Control | 3 (178) [1, 3, 8] | Yes | –102.5 [–157.3, –47.7] | 0% | 84 | 0.80 | Moderate | - Imprecision ☒  - Indirectness ☒  - Inconsistency ☐†  - Study limitations (ROB) ☒  - Publication bias ☒ |
| FA implant (0.5 µg/d) vs. Control | 2 (618) [14, 16] | Yes | –134.1 [–188.0, –80.1] | 50% | 70 | N/A | Low | - Imprecision ☒  - Indirectness ☒  - Inconsistency ☐†  - Study limitations (ROB) ☐‡  - Publication bias ☒ |
| FA implant (0.2 µg/d) vs. Control | 1 (560) [14] | N/A | –85.0 [–100.8, –69.2] | 0% | N/A | N/A | Moderate | Result was based on a randomized controlled trial with a large sample size |
| DEX implant (700 µg) *vs.* Control | 1 (701) [17] | N/A | –25.0 [–38.3, –11.7] | 0% | N/A | N/A | Moderate | Result was based on a randomized controlled trial with a large sample size |
| DEX implant (350 µg) *vs.* Control | 1 (697) [17] | N/A | –38.0 [–51.3, –24.7] | 0% | N/A | N/A | Moderate | Result was based on a randomized controlled trial with a large sample size |
| TA injection (≥ 8 mg) *vs.*  TA injection (4–8 mg) | 1 (43) [7] | N/A | –80.3 [–123.0, –37.6] | 100% | N/A | N/A | Very low | Result was based on a randomized controlled trial with small sample size |
| TA injection (4–8 mg) *vs.* TA injection (< 4 mg) | 3 (116) [4, 9, 21] | Yes | 5.4 [–37.6, 48.3] | 67% | 10 | 0.16 | Moderate | - Imprecision ☒  - Indirectness ☒  - Inconsistency ☒  - Study limitations (ROB) ☐‡  - Publication bias ☒ |
| TA injection (4–8 mg) vs.  DEX implant (700 µg) | 1 (29) [20] | N/A | –107.0 [–170.2, –43.8] | 100% | N/A | N/A | Very low | Result was based on a randomized controlled trial with a high risk of bias |
| FA implant (0.5 µg/d) vs.  FA implant (0.2 µg/d) | 2 (805) [12, 14] | Yes | –53.5 [–112.2, 5.2] | 0% | 82 | N/A | Moderate | - Imprecision ☒  - Indirectness ☒  - Inconsistency ☐†  - Study limitations (ROB) ☒  - Publication bias ☒ |
| DEX implant (700 µg) vs.  DEX implant (350 µg) | 1 (698) [17] | N/A | 13.0 [–0.3, 26.3] | 0% | N/A | N/A | Moderate | Result was based on a randomized controlled trial with a large sample size |

The mean difference and 95% CI were calculated using pairwise meta-analysis. The actual sample size is the sum of patients included in the body of evidence. The evidence quality was graded according to the Grading of Recommendations Assessment, Development, and Evaluation (GRADE) guidelines [23].

CMT, central macular thickness; CI, confident interval; ROB, risk of bias; TA, triamcinolone acetonide; N/A, not applicable; FA, fluocinolone acetonide; DEX, dexamethasone

* Required information size was evaluated according to GRADE guidelines 6 [25]. When calculating the required information size, we chose an α error of 0.05, β error of 0.2, a mean difference of 100 (implying a judgment that reductions in central macular thickness of more than 100 µm are important), and a standard deviation of 156 (according to two relevant studies) [14, 17]. It yields the corresponding required information size of 80

† Downgraded one level for inconsistency as a result of high statistical heterogeneity (I^2^ ≥ 40%) that could not be explained by subgroup analyses [27]

‡ Downgraded one level for high overall risk of bias within studies if the percentage of the studies with a high or unclear overall risk of bias within the body of evidence was ≥ 50% [22]. The overall risk of bias within individual studies was based on the assessment of the risks of bias for different domains (Table S2) [26]

☒ indicated no downgrading

☐ indicated that the quality of evidence was downgraded for the factor

**Table S10. Evaluation of the inconsistency between direct and indirect evidence for the effect of intravitreal corticosteroids on short-term CMT using node splitting model [28]**

| **Comparison** | **Direct evidence** | **Indirect evidence** | ***P* value** |
| --- | --- | --- | --- |
|  | **Mean difference of CMT (µm) change between two groups [95% CrI]** | |  |
| TA injection (4–8 mg) *vs.* Control | –100.0 [–170.0, –33.0] | –130.0 [–310.0, 44.0] | 0.70 |
| FA implant (0.2 µg/d) *vs.* Control | –85.0 [–190, –2.2] | –82.0 [–210.0, 47.0] | 0.97 |
| DEX implant (700 µg) *vs.* Control | –35.0 [–80.0, –1.7] | 5.1 [–100.0, 110.0] | 0.70 |
| TA injection (4–8 mg) *vs.*  DEX implant (700 µg) | –110.0 [–240.0, 23.0] | –78.0 [–210.0, 55.0] | 0.71 |

*CMT* central macular thickness; *CrI* credible interval; *TA* triamcinolone acetonide; *FA* fluocinolone acetonide; *DEX* dexamethasone

**Table S11. Estimates of effects and quality ratings for comparisons of intravitreal corticosteroids for increasing short-term IOP**

| **Comparison** | **Direct evidence*** | | **Indirect evidence†** | | **Network meta-analysis‡** | |
| --- | --- | --- | --- | --- | --- | --- |
|  | **Mean difference of IOP (mmHg) change between two groups [95% CI]** | **Quality** | **Mean difference of IOP (mmHg) change between two groups [95% CrI]** | **Quality** | **Mean difference of IOP (mmHg) change between two groups [95% CrI]** | **Quality** |
| TA injection (≥ 8 mg) *vs.* Control | 3.60 [1.60, 5.60] | Low | 0.02 [–4.60, 4.90] | Low | 2.08 [–1.05, 5.52] | Low |
| TA injection (4–8 mg) *vs.* Control | 1.30 [0.18, 2.42] | Low | 4.90 [–0.33, 9.90] | Low | 2.38 [–0.75, 5.70] | Low |
| TA injection (< 4 mg) *vs.* Control | - | - | 1.92 [–1.58, 5.64] | Low | 1.92 [–1.58, 5.64] | Low |
| TA injection (≥ 8 mg) *vs.*  TA injection (4–8 mg) | –1.60 [–2.89, –0.31] | Low | 2.30 [–3.20, 7.80] | Low | –0.31 [–3.22, 2.85] | Low |
| TA injection (≥ 8 mg) *vs.*  TA injection (< 4 mg) | –0.10 [–1.47, 1.27] | Low | 2.50 [–3.30, 8.40] | Low | 0.15 [–3.01, 3.49] | Low |
| TA injection (4–8 mg) *vs.* TA injection (< 4 mg) | 0.15 [–3.01, 3.49] | Low | 0.46 [–1.59, 2.48] | Low | 0.46 [–1.59, 2.48] | Low |

*IOP* intraocular pressure; *CI* confident interval; *CrI* credible interval; *TA* triamcinolone acetonide; *GRADE* Grading of Recommendations Assessment, Development, and Evaluation

* Direct evidence was based on pairwise meta-analysis which aggregates the results of the head-to-head comparisons between different treatments. Quality of direct evidence was assessed per the GRADE guidelines [23], with details of assessment presented in Table S12 in Additional files

† Indirect evidence was based on network meta-analysis which estimates the difference between two treatments based on their comparisons with other treatments in the network, but not their direct comparisons. Quality of indirect evidence was assessed per the GRADE guidelines for network meta-analysis [24]

‡ The quality of each network estimate was assessed per the GRADE guidelines for network meta-analysis [24]; it was determined by the quality of direct or indirect evidence whichever was higher

**Table S12. Quality of direct evidence for comparisons of intravitreal corticosteroids for increasing short-term IOP**

| **Comparison** | **Number of studies, (sample size)** | **Required information size met*** | **Mean difference of IOP (mmHg) between two groups [95% CI]** | **Overall ROB (high and unclear)** | **I^2^** | **Egger’s test**  **(P value)** | **Quality of evidence** | **Comments** |
| --- | --- | --- | --- | --- | --- | --- | --- | --- |
| TA injection (≥ 8 mg) *vs.* Control | 1 (40) [6] | N/A | 3.60 [1.60, 5.60] | 0% | N/A | N/A | Low | Result was based on a randomized controlled trial with small sample size |
| TA injection (4–8 mg) *vs.* Control | 1 (34) [3] | N/A | 1.30 [0.18, 2.42] | 0% | N/A | N/A | Low | Result was based on a randomized controlled trial with small sample size |
| TA injection (≥ 8 mg) *vs.*  TA injection (4–8 mg) | 1 (19) [2] | N/A | –1.60 [–2.89, –0.31] | 0% | N/A | N/A | Low | Result was based on a randomized controlled trial with small sample size |
| TA injection (≥ 8 mg) *vs.*  TA injection (< 4 mg) | 1 (17) [2] | N/A | –0.10 [–1.47, 1.27] | 0% | N/A | N/A | Low | Result was based on a randomized controlled trial with small sample size |
| TA injection (4–8 mg) *vs.* TA injection (< 4 mg) | 4 (119) [2, 4, 9, 19] | Yes | 0.15 [–3.01, 3.49] | 50% | 62 | 0.96 | Low | - Imprecision ☒  - Indirectness ☒  - Inconsistency ☐†  - Study limitations (ROB) ☐‡  - Publication bias ☒ |

The mean difference and 95% CI were calculated using pairwise meta-analysis. The actual sample size is the sum of patients included in the body of evidence. The evidence quality was graded according to the Grading of Recommendations Assessment, Development, and Evaluation (GRADE) guidelines [23].

*IOP* intraocular pressure; *CI* confident interval; *ROB* risk of bias; *TA* triamcinolone acetonide; *N/A* not applicable

* Required information size was evaluated according to GRADE guidelines 6 [25]. When calculating the required information size, we chose an α error of 0.05, β error of 0.2, a mean difference of 5 (implying a judgment that increase in intraocular pressure of more than 5 mmHg are important), and a standard deviation of 3 (according to three relevant studies) [2, 4, 9]. It yields the corresponding required information size of 14

† Downgraded one level for inconsistency as a result of high statistical heterogeneity (I^2^ ≥ 40%) that could not be explained by subgroup analyses

‡ ]Downgraded one level for high overall risk of bias within studies if the percentage of the studies with a high or unclear overall risk of bias within the body of evidence was ≥ 50% [22, 27]. The overall risk of bias within individual studies was based on the assessment of the risks of bias for different domains (Table S2) [26]

☒ indicated no downgrading

☐ indicated that the quality of evidence was downgraded for the factor

**Table S13. Evaluation of the inconsistency between direct and indirect evidence for the effect of intravitreal corticosteroids on short-term IOP using node splitting model [28]**

| **Comparison** | **Direct evidence** | **Indirect evidence** | ***P* value** |
| --- | --- | --- | --- |
|  | **Mean difference of IOP (mmHg) change between two groups [95% CrI]** | |  |
| TA injection (≥ 8 mg) *vs.* Control | 3.60 [–0.21, 7.30] | 0.02 [–4.60, 4.90] | 0.19 |
| TA injection (4–8 mg) *vs.* Control | 1.30 [–2.30, 4.90] | 4.90 [–0.33, 9.90] | 0.19 |
| TA injection (≥ 8 mg) *vs.*  TA injection (4–8 mg) | –1.60 [–5.20, 2.10] | 2.30 [–3.20, 7.80] | 0.16 |
| TA injection (≥ 8 mg) *vs.*  TA injection (< 4 mg) | –0.10 [–3.80, 3.70] | 2.50 [–3.30, 8.40] | 0.33 |

*IOP* intraocular pressure; *CrI* credible interval; *TA* triamcinolone acetonide

**Figure S1. Pooled risk of bias**

**
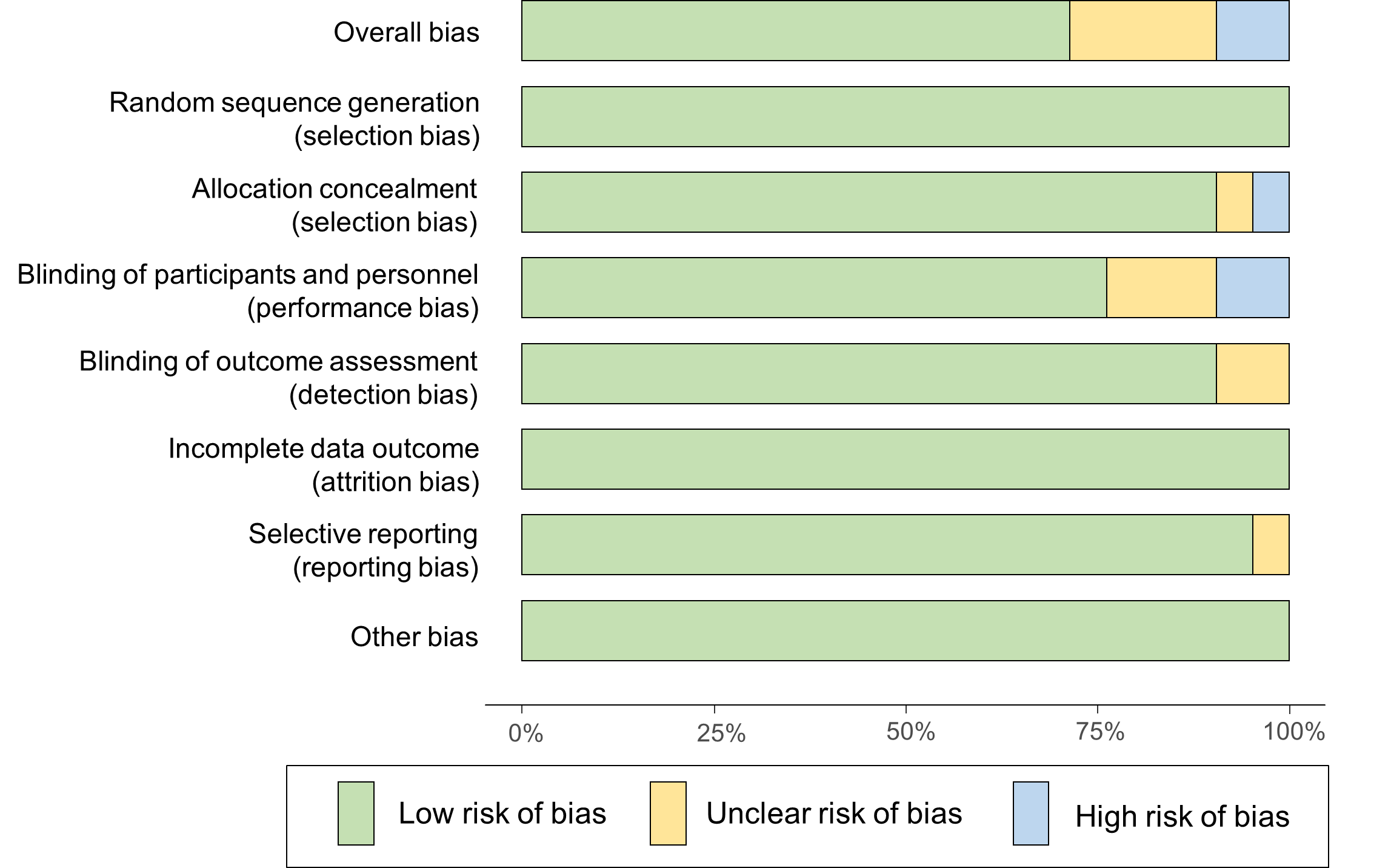
**

**Figure S2. Network geometry of different intravitreal corticosteroids to reduce short-term CMT.** CMT, central macular thickness; TA, triamcinolone acetonide; FA, fluocinolone acetonide; DEX, dexamethasone.


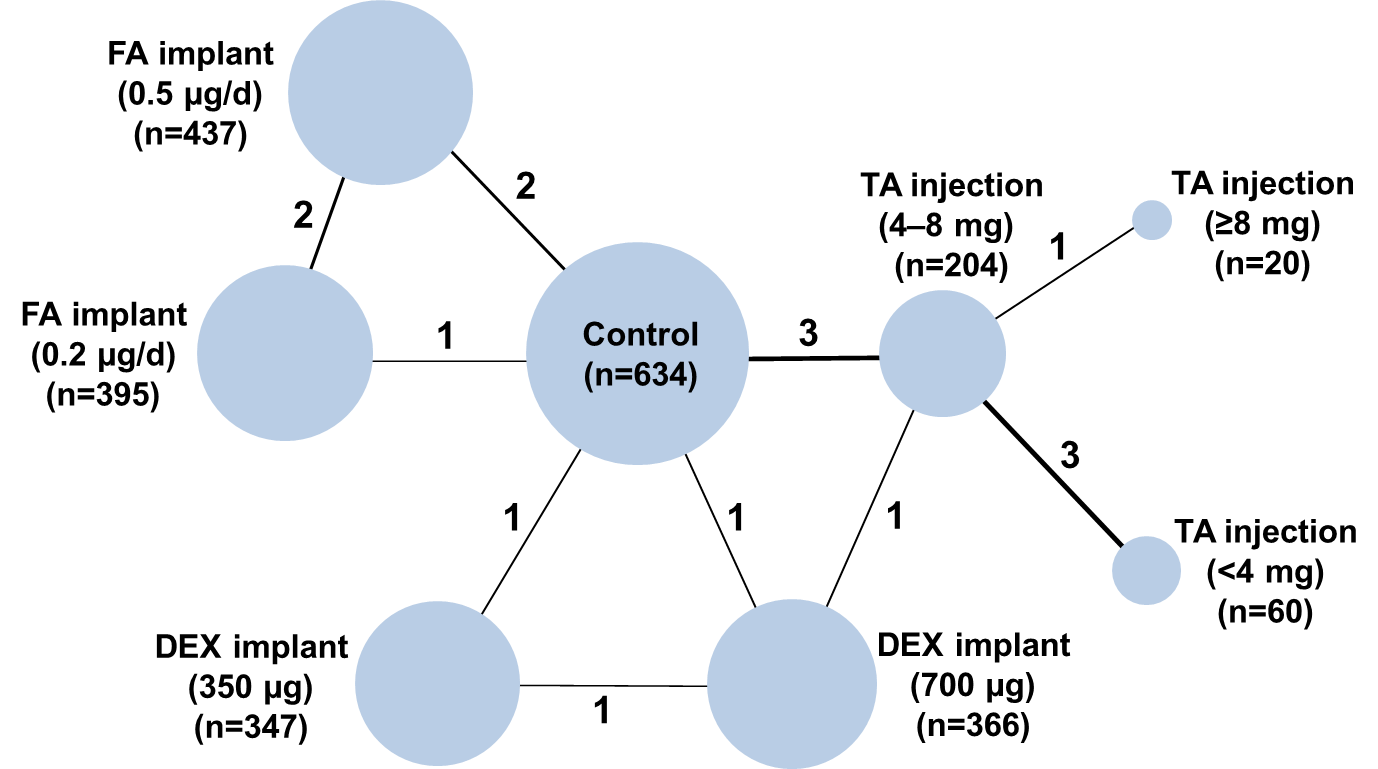


**Figure S3. Effects of the different intravitreal corticosteroids on short-term CMT.** CMT, central macular thickness; CrI, credible interval; TA, triamcinolone acetonide; FA, fluocinolone acetonide; DEX, dexamethasone.


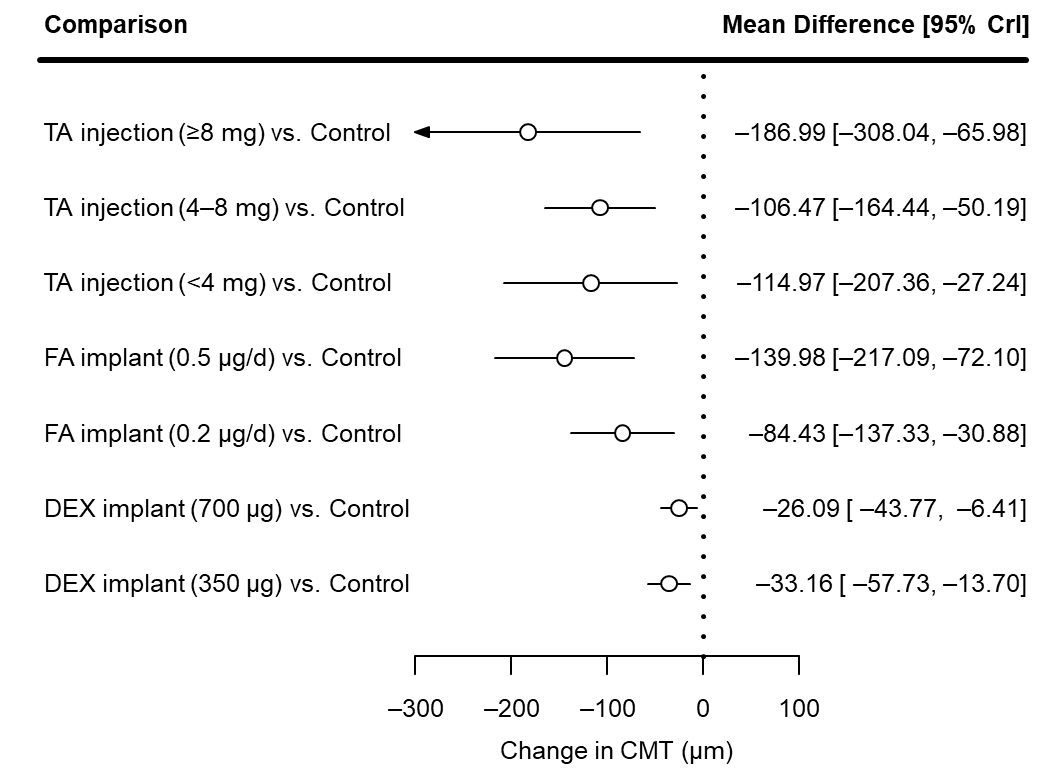


**Figure S4. SUCRA scores for the effects of different intravitreal corticosteroids on short-term CMT.** SUCRA, surface under the cumulative ranking curve; CMT, central macular thickness; TA, triamcinolone acetonide; FA, fluocinolone acetonide; DEX, dexamethasone.


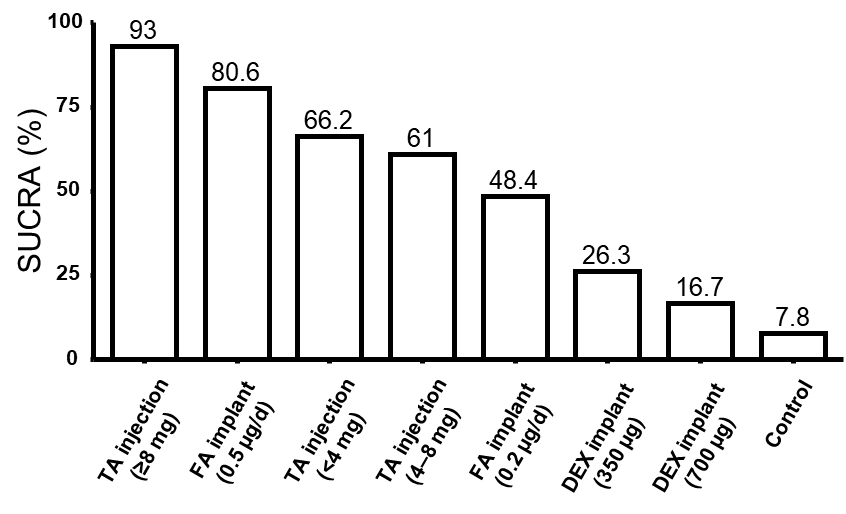


**Figure S5. Network geometry of different intravitreal corticosteroids for increasing short-term IOP.** IOP, intraocular pressure; TA, triamcinolone acetonide.


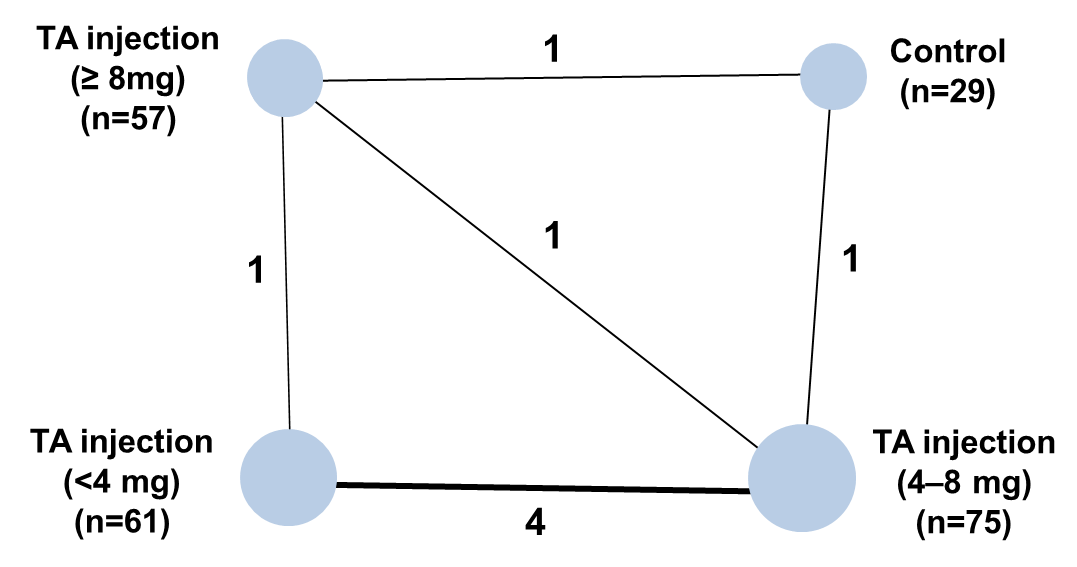


**Figure S6. Effects of the different intravitreal corticosteroids on short-term IOP.** IOP, intraocular pressure; CrI, credible interval; TA, triamcinolone acetonide.


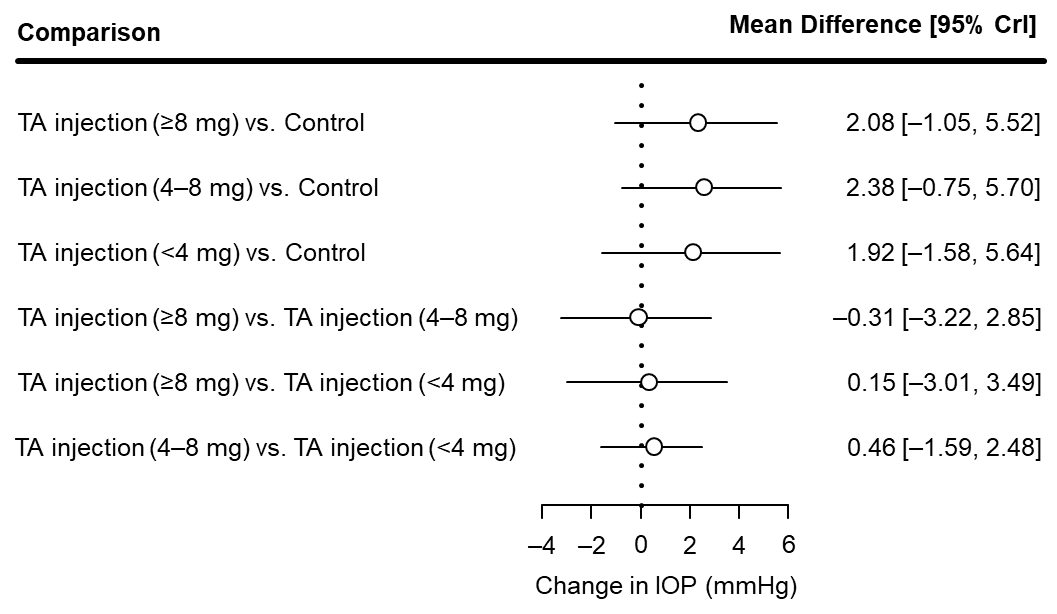


**Appendix S1. Search strategies**

**Database: Ovid MEDLINE(R) ALL**

1946 to November 10, 2020

| **Number** | **Search Concept** | **Results** |
| --- | --- | --- |
| 1 | exp diabetic retinopathy/ | 24780 |
| 2 | exp macular edema cystoid/ | 7182 |
| 3 | exp macular degeneration/ | 24501 |
| 4 | (macula$ adj2 edema).tw. | 10142 |
| 5 | (macula$ adj2 oedema).tw. | 2048 |
| 6 | DME.tw. | 3511 |
| 7 | DMO.tw. | 705 |
| 8 | CME.tw. | 6689 |
| 9 | CSME.tw. | 221 |
| 10 | (macula$ adj2 swell$).tw. | 40 |
| 11 | microaneurysm$.tw. | 1562 |
| 12 | (dilat$ adj2 capillar$).tw. | 870 |
| 13 | or/1-12 | 60159 |
| 14 | exp steroids/ | 860161 |
| 15 | exp triamcinolone/ | 9467 |
| 16 | triamcin$.tw. | 7833 |
| 17 | (steroid$ or glucocorticoid$).tw. | 291978 |
| 18 | hydrocortisone$.tw. | 16402 |
| 19 | prednisolone$.tw. | 26221 |
| 20 | dexamethasone$.tw. | 56982 |
| 21 | fluorometholone$.tw. | 290 |
| 22 | or/14-21 | 1047482 |
| 23 | randomized controlled trial.pt. | 516819 |
| 24 | controlled clinical trial.pt. | 93920 |
| 25 | (randomized or randomly or trial or groups or placebo).ti,ab. | 2990159 |
| 26 | or/23-25 | 3121603 |
| 27 | 13 and 22 and 26 | 1162 |

**Database: Embase**

1974 to November 10, 2020

| **Number** | **Search Concept** | **Results** |
| --- | --- | --- |
| 1 | exp diabetic retinopathy/ | 43908 |
| 2 | exp retina macula cystoid edema/ | 5614 |
| 3 | exp retina macula degeneration/ | 17775 |
| 4 | (macula$ adj2 edema).tw. | 14384 |
| 5 | (macula$ adj2 oedema).tw. | 2798 |
| 6 | DME.tw. | 5260 |
| 7 | DMO.tw. | 825 |
| 8 | CME.tw. | 12486 |
| 9 | CSME.tw. | 314 |
| 10 | (macula$ adj2 swell$).tw. | 56 |
| 11 | microaneurysm$.tw. | 2123 |
| 12 | (dilat$ adj2 capillar$).tw. | 1354 |
| 13 | or/1-12 | 89730 |
| 14 | exp steroids/ | 1520461 |
| 15 | exp triamcinolone/ | 14406 |
| 16 | triamcin$.tw. | 10028 |
| 17 | (steroid$ or glucocorticoid$).tw. | 400160 |
| 18 | hydrocortisone$.tw. | 19155 |
| 19 | prednisolone$.tw. | 39141 |
| 20 | dexamethasone$.tw. | 78654 |
| 21 | fluorometholone$.tw. | 394 |
| 22 | or/14-21 | 1634439 |
| 23 | (randomized or randomly or trial or groups or placebo).ti,ab. | 4181468 |
| 24 | exp randomized controlled trial | 633324 |
| 25 | 23 or 24 | 4265691 |
| 26 | 13 and 22 and 25 | 1784 |

**Web of Science: Core Collection**

Science Citation Index expanded: 1900-present

Social Sciences Citation Index: 1900-present

| **Number** | **Search Concept** | **Results** |
| --- | --- | --- |
| 1 | TS= (“diabetic retinopathy” OR “retina macula cystoid edema” OR “retina macula degeneration” OR DME OR DMO OR CME OR CSME OR microaneurysm) | 55614 |
| 2 | TS=((macula) NEAR/2 (edema OR oedema OR swell)) | 312 |
| 3 | #2 OR #1 | 55764 |
| 4 | TS= (steroids OR triamcinolone OR glucocorticoid OR hydrocortisone OR prednisolone OR dexamethasone OR fluorometholone) | 612749 |
| 5 | TS=(randomized or randomly or trial or groups OR placebo) | 6283463 |
| 6 | #5 AND #4 AND #3 | 1582 |

**Cochrane Library**

| **Number** | **Search Concept** | **Results** |
| --- | --- | --- |
| 1 | Diabetic Retinopathy | 4232 |
| 2 | macular edema cystoid | 536 |
| 3 | macular degeneration | 3351 |
| 4 | macula* near edema | 3436 |
| 5 | macula* near oedema | 3436 |
| 6 | DME OR DMO OR CME OR CSME | 3150 |
| 7 | macula* near swell* | 39 |
| 8 | microaneurysm* | 152 |
| 9 | dilat* near capillar* | 54 |
| 10 | (#1 OR #2 OR #3 OR #4 OR #5 OR #6 OR #7 OR #8 OR #9) | 11100 |
| 11 | Steroids | 11090 |
| 12 | triamcinolone | 3283 |
| 13 | triamcin* | 3348 |
| 14 | hydrocortisone | 9518 |
| 15 | prednisolone | 7528 |
| 16 | dexamethasone | 12072 |
| 17 | fluorometholone | 211 |
| 18 | steroid* or glucocorticoid* | 39411 |
| 19 | (#11 OR #12 OR #13 OR #14 OR #15 OR #16 OR #17 OR #18) | 62010 |
| 20 | (#10 AND #19) | 1548 |
| 21 | #20 in trials | 1456 |

**References**

1. Sutter FK, Simpson JM, Gillies MC. **Intravitreal triamcinolone for diabetic macular edema that persists after laser treatment: three-month efficacy and safety results of a prospective, randomized, double-masked, placebo-controlled clinical trial**. Ophthalmology. 2004;**111**(11):2044-9.

2. Spandau UH, Derse M, Schmitz-Valckenberg P, Papoulis C, Jonas JB. **Dosage dependency of intravitreal triamcinolone acetonide as treatment for diabetic macular oedema**. Br J Ophthalmol. 2005;**89**(8):999-1003.

3. Audren F, Erginay A, Haouchine B, Benosman R, Conrath J, Bergmann JF, et al. **Intravitreal triamcinolone acetonide for diffuse diabetic macular oedema: 6-month results of a prospective controlled trial**. Acta Ophthalmol Scand. 2006;**84**(5):624-30.

4. Audren F, Lecleire-Collet A, Erginay A, Haouchine B, Benosman R, Bergmann JF, et al. **Intravitreal triamcinolone acetonide for diffuse diabetic macular edema: phase 2 trial comparing 4 mg vs 2 mg**. Am J Ophthalmol. 2006;**142**(5):794-9.

5. Gillies MC, Sutter FK, Simpson JM, Larsson J, Ali H, Zhu M. **Intravitreal triamcinolone for refractory diabetic macular edema: two-year results of a double-masked, placebo-controlled, randomized clinical trial**. Ophthalmology. 2006;**113**(9):1533-8.

6. Jonas JB, Kamppeter BA, Harder B, Vossmerbaeumer U, Sauder G, Spandau UH. **Intravitreal triamcinolone acetonide for diabetic macular edema: a prospective, randomized study**. J Ocul Pharmacol Ther. 2006;**22**(3):200-7.

7. Lam DS, Chan CK, Mohamed S, Lai TY, Li KK, Li PS, et al. **A prospective randomised trial of different doses of intravitreal triamcinolone for diabetic macular oedema**. Br J Ophthalmol. 2007;**91**(2):199-203.

8. Dehghan MH, Ahmadieh H, Ramezani A, Entezari M, Anisian A. **A randomized, placebo-controlled clinical trial of intravitreal triamcinolone for refractory diabetic macular edema**. Int Ophthalmol. 2008;**28**(1):7-17.

9. Hauser D, Bukelman A, Pokroy R, Katz H, Len A, Thein R, et al. **Intravitreal triamcinolone for diabetic macular edema - Comparison of 1, 2, and 4 mg**. Retina. 2008;**28**(6):825-30.

10. Kim JE, Pollack JS, Miller DG, Mittra RA, Spaide RF. **ISIS-DME: A prospective, randomized, dose-escalation intravitreal steroid injection study for refractory diabetic macular edema**. Retina. 2008;**28**(5):735-40.

11. Larsson J, Kifley A, Zhu M, Wang JJ, Mitchell P, Sutter FK, et al. **Rapid reduction of hard exudates in eyes with diabetic retinopathy after intravitreal triamcinolone: data from a randomized, placebo-controlled, clinical trial**. Acta Ophthalmol. 2009;**87**(3):275-80.

12. Campochiaro PA, Hafiz G, Shah SM, Bloom S, Brown DM, Busquets M, et al. **Sustained ocular delivery of fluocinolone acetonide by an intravitreal insert**. Ophthalmology. 2010;**117**(7):1393-9.e1393.

13. Chan CK, Mohamed S, Lee VY, Lai TY, Shanmugam MP, Lam DS. **Intravitreal dexamethasone for diabetic macular edema: a pilot study**. Ophthalmic Surg Lasers Imaging. 2010;**41**(1):26-30.

14. Campochiaro PA, Brown DM, Pearson A, Ciulla T, Boyer D, Holz FG, et al. **Long-term benefit of sustained-delivery fluocinolone acetonide vitreous inserts for diabetic macular edema**. Ophthalmology. 2011;**118**(4):626-35.e622.

15. Campochiaro PA, Brown DM, Pearson A, Chen S, Boyer D, Ruiz-Moreno J, et al. **Sustained delivery fluocinolone acetonide vitreous inserts provide benefit for at least 3 years in patients with diabetic macular edema**. Ophthalmology. 2012;**119**(10):2125-32.

16. Pearson PA, Comstock TL, Ip M, Callanan D, Morse LS, Ashton P, et al. **Fluocinolone acetonide intravitreal implant for diabetic macular edema: a 3-year multicenter, randomized, controlled clinical trial**. Ophthalmology. 2011;**118**(8):1580-7.

17. Boyer DS, Yoon YH, Belfort R, Bandello F, Maturi RK, Augustin AJ, et al. **Three-year, randomized, sham-controlled trial of dexamethasone intravitreal implant in patients with diabetic macular edema**. Ophthalmology. 2014;**121**(10):1904-14.

18. Danis RP, Sadda S, Li XY, Cui H, Hashad Y, Whitcup SM. **Anatomical effects of dexamethasone intravitreal implant in diabetic macular oedema: a pooled analysis of 3-year phase III trials**. Br J Ophthalmol. 2016;**100**(6):796-801.

19. Lodhi SAK, Reddy S, Devulapally S. **Intravitreal triamcinolone in diabetic macular edema: a comparative study of 1mg and 4mg doses**. Journal of Evolution of Medical and Dental Sciences-Jemds. 2015;**4**(53):9191-201.

20. Mylonas G, Georgopoulos M, Malamos P, Georgalas I, Koutsandrea C, Brouzas D, et al. **Comparison of dexamethasone intravitreal implant with conventional triamcinolone in patients with postoperative cystoid macular edema**. Curr Eye Res. 2017;42(4):648-52.

21. Zhou H. **Comparison between 2mg and 4mg intravitreal triamcinolone acetonide combined with laser photocoagulation for diabetic macular edema**. International eye science. 2016;16(9):1692-4.

22. Higgins JP, Green S. **Cochrane handbook for systematic reviews of interventions**. vol. 4: John Wiley & Sons; 2011.

23. Guyatt G, Oxman AD, Akl EA, Kunz R, Vist G, Brozek J, et al. **GRADE guidelines: 1. Introduction-GRADE evidence profiles and summary of findings tables**. J Clin Epidemiol. 2011;**64**(4):383-94.

24. Puhan MA, Schünemann HJ, Murad MH, Li T, Brignardello-Petersen R, Singh JA, et al. **A GRADE Working Group approach for rating the quality of treatment effect estimates from network meta-analysis**. BMJ. 2014;**349**:g5630.

25. Guyatt GH, Oxman AD, Kunz R, Brozek J, Alonso-Coello P, Rind D, et al. **GRADE guidelines 6. Rating the quality of evidence—imprecision**. J Clin Epidemiol. 2011;**64**(12):1283-93.

26. Higgins JP, Altman DG, Gøtzsche PC, Jüni P, Moher D, Oxman AD, et al. **The Cochrane Collaboration’s tool for assessing risk of bias in randomised trials**. BMJ. 2011;**343**:d5928.

27. Guyatt GH, Oxman AD, Kunz R, Woodcock J, Brozek J, Helfand M, et al. **GRADE guidelines: 7. Rating the quality of evidence—inconsistency**. J Clin Epidemiol. 2011;**64**(12):1294-302.

28. Dias S, Welton NJ, Caldwell DM, Ades AE. **Checking consistency in mixed treatment comparison meta-analysis**. Stat Med. 2010;**29**(7-8):932-44.
